# Integrated Transcriptomic Analysis of Skin Pigmentation Development in *Amphiprion frenatus*

**DOI:** 10.3390/ijms27188289

**Published:** 2026-09-17

**Authors:** Kuang-Den Chen, Mong-Fong Lee, Ming-Chung Cheng, Yuan-Shing Ho, Winton Cheng, Ta-Chien Tseng, En-Lieng Lau

**Affiliations:** 1 Institute for Translational Research in Biomedicine, Kaohsiung Chang Gung Memorial Hospital, Kaohsiung 833401, Taiwan; 2 Liver Transplantation Center, Department of General Surgery, Kaohsiung Chang Gung Memorial Hospital, Kaohsiung 833401, Taiwan; 3 Department of Aquaculture, National Penghu University of Science and Technology, Pingtung 880011, Taiwan; 4 Eastern Fishery Research Center, Fisheries Research Institute, Taitung 961006, Taiwan; 5 Department of Aquaculture, National Pingtung University of Science and Technology, Pingtung 912301, Taiwan; 6 Institute of Biotechnology, National Tsing-Hua University, Hsinchu 300044, Taiwan; 7 Freshwater Aquaculture Research Center, Fisheries Research Institute, Changhua 505031, Taiwan

**Keywords:** *Amphiprion frenatus*, pigmentation, skin development, transcriptomics, translatomics, chromatophores

## Abstract

Skin pigmentation in teleosts is a complex process governed by molecular and cellular mechanisms during development. In this study, we investigated the pigmentation process in the tomato clownfish (*Amphiprion frenatus*) using an integrated approach combining histological analysis and transcriptomics (RNA-Seq and Ribo-Seq). Histological examination revealed the stepwise differentiation of chromatophores during post-embryonic development. Transcriptomic profiling at 14 days post-hatching (dph) identified extensive changes in transcript abundance, with 2063 of 2667 differentially expressed unigenes (DEUs) upregulated relative to hatching. These genes predominantly governed innate defense, digestion, and early pigmentary development, driven by the enrichment of Gene Ontology (GO) terms including inflammatory response, digestion, and developmental pigmentation. Integration of transcriptomic and Ribo-Seq datasets identified widespread discordance between transcript abundance and ribosome-protected fragment (RPF) abundance. These discordant expression patterns suggest candidate post-transcriptional regulatory events during skin pigmentation development. Quantitative PCR analysis at 23 and 45 dph confirmed the dynamic upregulation of genes involved in cell proliferation (*myc*, *pcna*), pigment transport (*myo5a*, *rab38*), and pigment cell signaling (*mchr1*). These findings suggest that skin pigmentation is associated with the selected transcript-abundance differences in *A*. *frenatus*. This study offers new insights into the molecular basis of chromatophore development and pigment pattern formation in coral reef fish species.

## 1. Introduction

The skin is the largest organ in teleost fishes and functions as the primary interface between the organism and its aquatic environment, contributing to mechanical protection, osmoregulation, innate immunity, and sensory perception [1,2]. During postembryonic development, the integument undergoes extensive epidermal stratification, dermal differentiation, extracellular matrix remodeling, basement membrane maturation, and scale formation [3,4]. These coordinated developmental processes establish the structural foundation for pigment cell organization and ultimately determine adult skin coloration [5]. Skin pigmentation contributes to camouflage, species recognition, predator avoidance, and sexual selection [6]. Consequently, teleost skin has emerged as an excellent vertebrate model for investigating organ morphogenesis, epithelial–mesenchymal interactions, and developmental pattern formation.

Teleost pigment cells originate primarily from neural crest-derived progenitors [7]. These cells differentiate into multiple chromatophore classes, including melanophores, xanthophores, iridophores, erythrophores, leucophores, and, in some species, cyanophores [7,8]. Rather than functioning independently, chromatophores communicate through direct cellular interactions that regulate migration, survival, and spatial organization [9]. Recent single-cell and developmental studies further demonstrate that pigment pattern formation depends on interactions between chromatophores and surrounding skin tissues [10]. Recent studies have demonstrated that pigment pattern formation is a dynamic developmental process governed by genetic, endocrine, and environmental cues rather than by pigment synthesis alone [1,11]. Advances in developmental genetics, live imaging, and transcriptomic approaches have substantially improved our understanding of chromatophore lineage specification and tissue organization [4,9], revealing that pigment pattern formation involves complex regulatory networks integrating multiple cell types throughout skin development [12,13]. Nevertheless, the molecular mechanisms coordinating chromatophore differentiation with epidermal and dermal morphogenesis remain incompletely understood, particularly in marine teleosts [11,14].

Among marine teleosts, clownfishes (*Amphiprion* spp.) have emerged as one of the most informative experimental systems for investigating vertebrate metamorphosis, pigment pattern formation, and evolutionary developmental biology. Over the past decade, pioneering studies have transformed clownfishes from classical ecological models into powerful developmental model organisms. Roux et al. established the first standardized postembryonic developmental staging system for clownfish, providing a common framework for developmental studies [15]. Holzer et al. demonstrated that thyroid hormones function as major endocrine regulators of clownfish metamorphosis [16]. Salis et al. subsequently showed that thyroid hormones regulate the formation and environmental plasticity of the characteristic white bars [17]. Transcriptomic analyses further identified endocrine pathways coordinating multiple developmental processes during metamorphosis [18]. Together, these studies established the endocrine and developmental basis of clownfish pigmentation and identified chromatophore differentiation as a central event during juvenile color pattern formation. More recently, transcriptomic and developmental analyses have continued to expand this framework by demonstrating how endocrine signaling coordinates tissue differentiation throughout metamorphosis [19,20]. Despite these important advances, the molecular mechanisms linking transcriptional regulation, translational regulation, and skin morphogenesis during clownfish pigmentation remain largely unexplored.

High-throughput RNA sequencing (RNA-Seq) has become an indispensable tool for investigating developmental processes and pigment formation in teleost fishes by enabling genome-wide characterization of gene expression [21,22]. However, transcript abundance alone frequently provides an incomplete representation of cellular activity because protein production is extensively regulated after transcription. Post-transcriptional mechanisms—including mRNA stability, alternative splicing, microRNA-mediated regulation, ribosome recruitment, upstream open reading frames, and translational efficiency—can substantially modify gene expression outputs without corresponding changes in transcript abundance [23,24]. Consequently, mRNA levels often exhibit only moderate correlation with protein abundance, particularly during rapid developmental transitions. Ribosome profiling (Ribo-Seq), which sequences ribosome-protected mRNA fragments, enables genome-wide identification of actively translated transcripts and quantitative assessment of translational efficiency at codon-level resolution [25,26]. Integrating RNA-Seq with Ribo-Seq therefore provides a comprehensive framework for simultaneously evaluating transcriptional and translational regulation, allowing genes under differential translational control to be identified and providing biological insights that cannot be obtained from transcriptomic analyses alone [27,28]. Although integrated transcriptomic and translatomic approaches have increasingly been applied in developmental biology, their application to teleost skin development and pigment pattern formation remains remarkably limited. Most previous studies have focused primarily on transcriptional profiling or individual signaling pathways, while relatively few have examined how transcriptional and translational regulation are coordinated during the rapid skin remodeling that accompanies metamorphosis.

In the present study, we combined histological analysis, scanning electron microscopy (SEM), RNA sequencing, ribosome profiling, and quantitative real-time PCR (qPCR) to investigate the molecular mechanisms underlying skin development and pigmentation in *A. frenatus*. By comparing developmental stages before and after the establishment of juvenile pigmentation (23 and 45 dph), we characterized structural changes in the skin together with transcriptional and post-transcriptional dynamics associated with epidermal maturation, dermal development, chromatophore differentiation, and pigment formation. Our integrated analyses identified differentially expressed genes and genes exhibiting discordant RNA and RPF abundance involved in skin morphogenesis and pigmentation, providing new insights into the multilayered regulatory mechanisms governing teleost skin development and establishing a valuable molecular resource for future studies of chromatophore biology, developmental evolution, and pigmentation in non-model marine fishes.

## 2. Results

### 2.1. Morphological Development and Pigmentation Progression in A. frenatus

**Figure 1:**
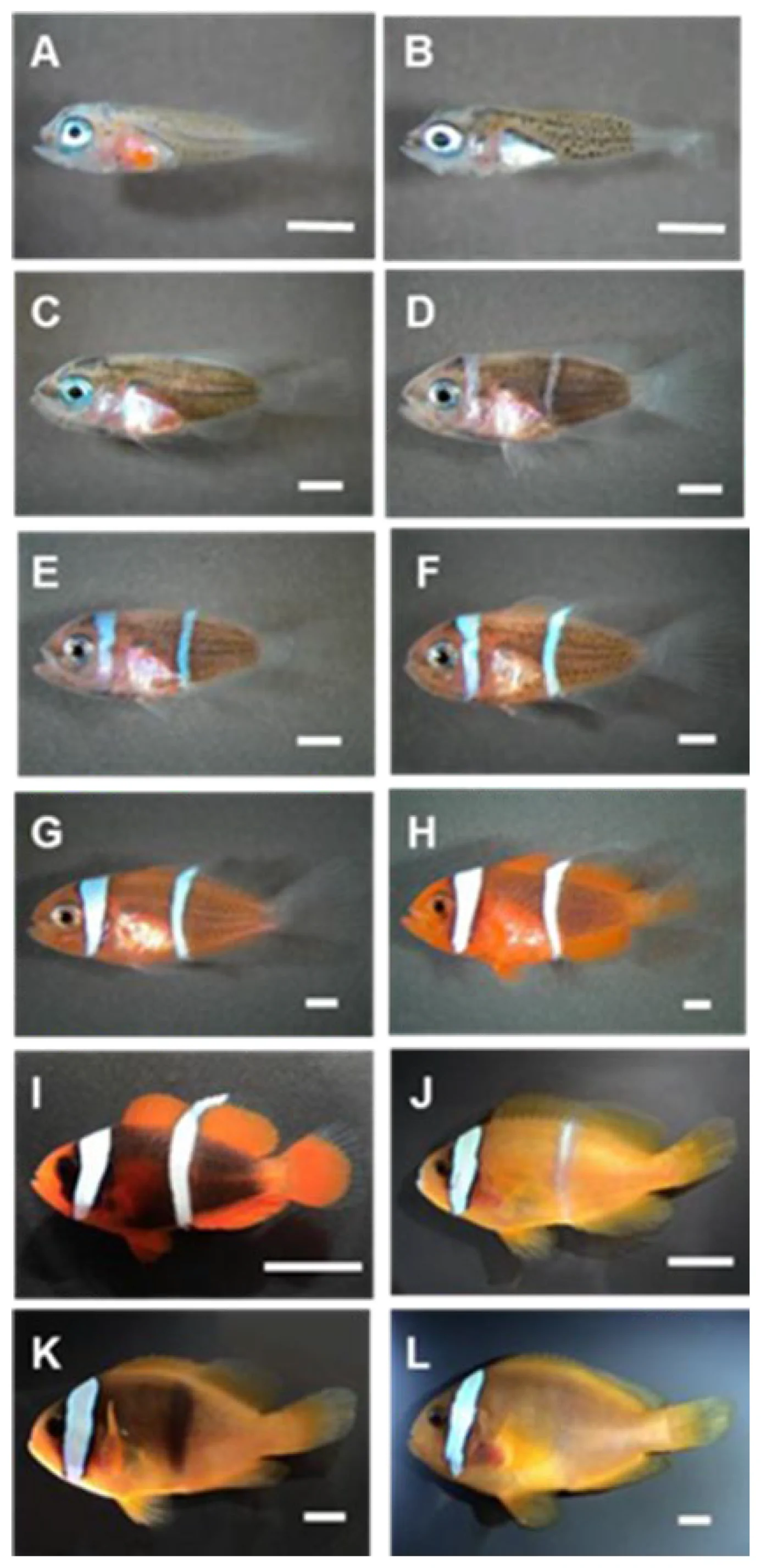
Morphological development and pigmentation progression in *A. frenatus* from larval to juvenile stages. (**A**) Newly hatched larva (0 dph) showing a transparent body, undeveloped fins, and no pigmentation. (**B**) At 1 dph, partial yolk absorption is evident, with the body remaining largely transparent. (**C**) At 5 dph, initial melanophore development appears along the dorsal and caudal regions. (**D**) At 10 dph, dark pigmentation continues, resulting in a slight grayish tint. (**E**) At 12 dph, body pigmentation darkens, and fin structures become more defined. (**F**) At 14 dph, brownish pigmentation is apparent along the trunk, and the eyes darken. (**G**) At 20 dph, orange pigmentation begins to emerge in the posterior body. (**H**) At 23 dph, orange coloration becomes more distinct, especially in the posterior body and fins. (**I**) At 45 dph, the first white vertical bar appears on the head, accompanied by intensified pigmentation. (**J**) At 90 dph, the orange hue dominates the body, and the head bar is well-defined. (**K**) At 140 dph, the body exhibits a deep orange-red coloration with a stable bar pattern. (**L**) At 240 dph, juveniles display the species’ mature phenotype, characterized by a deep reddish-orange body and a prominent white head bar. Scale bars: A–H = 1 mm; I–L = 5 mm.

The morphological development and pigmentation progression of *A*. *frenatus* from the larval to juvenile stages were documented through imaging (Figure 1). At 0 dph, newly hatched larvae exhibited a wholly transparent body with minimal structural differentiation and no apparent pigmentation (Figure 1A). By 1 dph, yolk absorption had initiated while body transparency persisted (Figure 1B). At 5 dph, the earliest signs of melanophore accumulation appeared, primarily along the dorsal and caudal regions (Figure 1C), which intensified by 10 dph, giving the body a faint grayish tone (Figure 1D). By 12 dph, the body had darkened further. The fin structures became more distinct (Figure 1E). At 14 dph, a brownish hue emerged along the trunk, and eye pigmentation increased (Figure 1F). The appearance of an orange coloration was first evident at 20 dph, beginning in the posterior body (Figure 1G), and became more pronounced throughout the fins and body by 23 dph (Figure 1H). At 45 dph, the first white vertical head bar—a hallmark of the species—was observed (Figure 1I). As development continued, the orange pigmentation became dominant, and the white bar was well defined by 90 dph (Figure 1J). By 140 dph, individuals exhibited a deep orange-red body coloration with stabilized bar patterns (Figure 1K). By 240 dph, juveniles displayed the fully mature coloration typical of the species, characterized by deep orange pigmentation and a distinct white head bar (Figure 1L). These observations indicate a sequential and coordinated pattern of pigment cell differentiation and regional pigmentation during early development.

### 2.2. Histological Development of the Skin in A. frenatus

**Figure 2:**
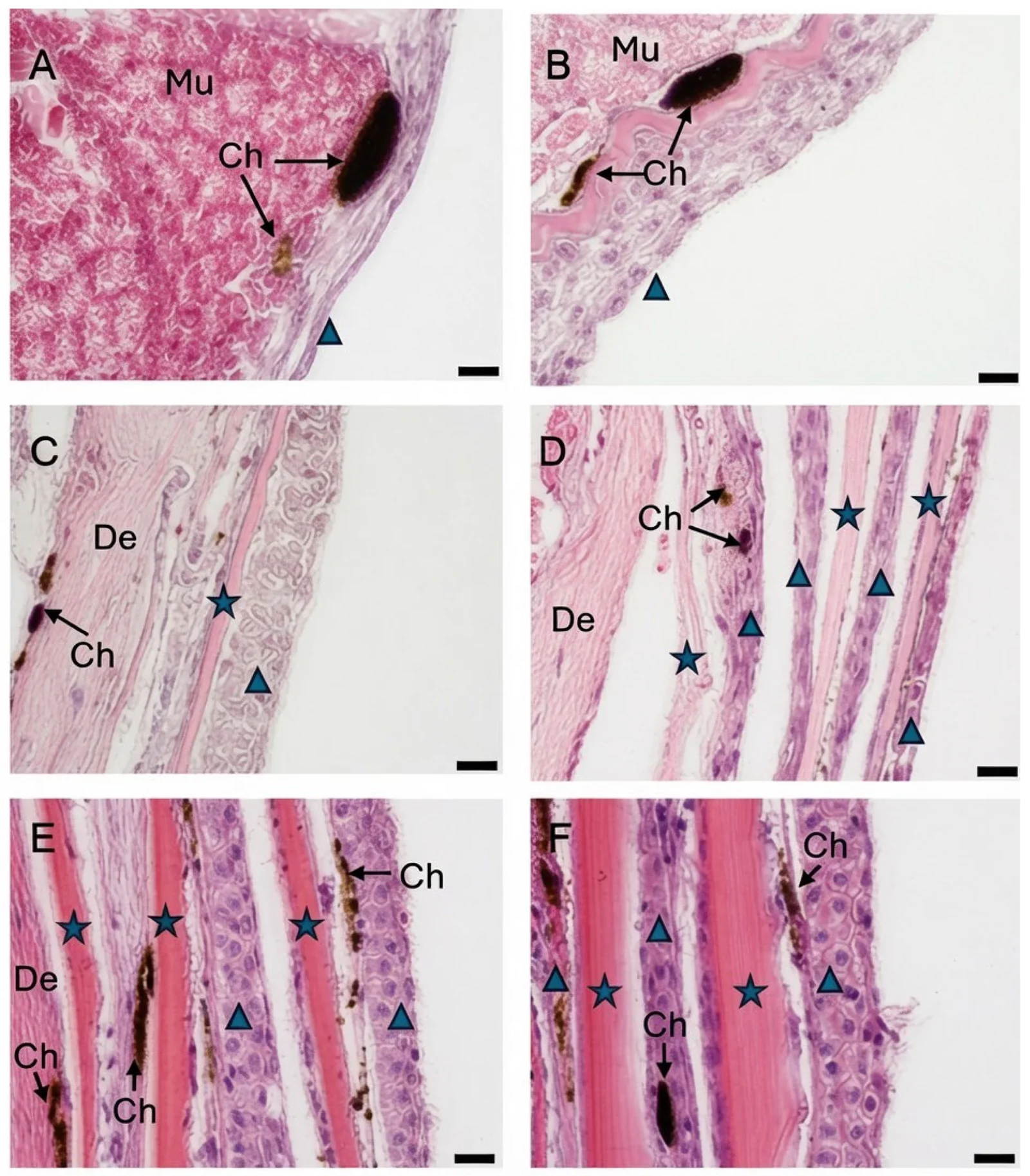
Histological development of the skin in *A. frenatus* from 14 to 240 dph. Representative H&E-stained longitudinal sections showing progressive skin maturation at (**A**) 14, (**B**) 23, (**C**) 45, (**D**) 90, (**E**) 140, and (**F**) 240 dph. At 14 and 23 dph, chromatophores (Ch) were observed beneath the thin epidermis (blue arrowheads) and near the developing dermo-epidermal interface. At later stages, increasing numbers of chromatophores were distributed within the superficial dermal region. A distinct dermis (De) and the first scale primordia (blue stars) become evident at 45 dph. At 90 dph, epidermal thickening, dermal expansion, and scale development are apparent, together with an increased number of chromatophores. At 140 and 240 dph, the skin displays a multilayered epidermis, a well-developed dermis containing overlapping scales, and abundant chromatophores. Black arrows indicate chromatophores; blue arrowheads indicate the epidermis; blue stars indicate scale primordia (**C**) and developing scales (**D**–**F**). Scale bars = 10 μm.

Histological examination revealed progressive maturation of the skin in *A*. *frenatus* from 14 to 240 dph (Figure 2). At 14 dph (Figure 2A), the skin consisted of a thin epidermis overlying the musculature, with a small number of chromatophores located beneath the epidermis. A similar histological organization was observed at 23 dph (Figure 2B), although the chromatophores became more conspicuous. By 45 dph (Figure 2C), a distinct dermis and the first scale primordia were evident, indicating the onset of dermal differentiation and scale formation. At 90 dph (Figure 2D), the epidermis became thicker, accompanied by dermal expansion, further scale development, and an increased abundance of chromatophores. At 140 dph (Figure 2E), the epidermis had become multilayered, while the dermis contained well-developed scales and numerous chromatophores. These histological features became more pronounced at 240 dph (Figure 2F), with the skin exhibiting a mature organization characterized by a multilayered epidermis, a well-developed dermis containing overlapping scales, and abundant chromatophores distributed throughout the dermal region. Together, these observations demonstrate a coordinated progression of epidermal stratification, dermal differentiation, scale development, and chromatophore accumulation during post-embryonic skin maturation in *A. frenatus*.

### 2.3. Scanning Electron Microscopy (SEM) of Skin Structure

**Figure 3:**
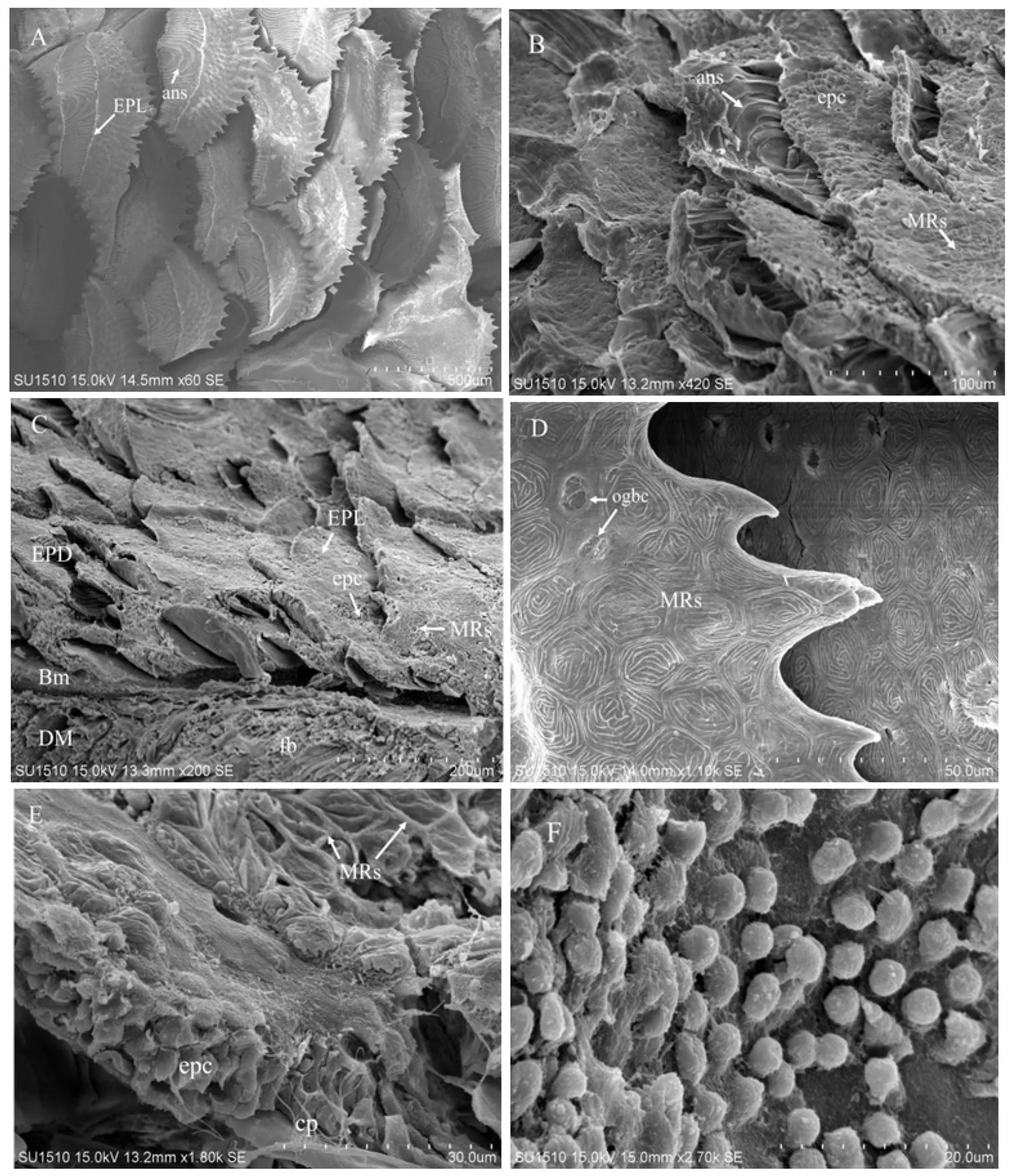
Scanning electron micrographs of the skin-surface architecture of juvenile *A*. *frenatus*. (**A**) Scales with ctenii at the posterior ends were arranged in an overlapping manner. Parts of the epithelial layers (EPL) on the scales were removed during preparation, thereby revealing the annulus (ans). (**B**) Ans, epithelial cells (EPCs) and microridges (MRs) were observed on the damaged surface of the epidermis (EPD). (**C**) The lateral view of the trunk showed EPD with overlapping scales, covered by EPL and dermis (DM), containing fibroblasts (fb). The basement membrane (BM) was located between EPD and DM. (**D**) The intact skin surface showed clear MRs at the apex of the EPD. Putative apical openings of goblet cells (ogbc) were visible between MRs. (**E**) EPD contained apical MRs, stratified EPC, and capillary (cp) from blood vessels in DM. (**F**) The lowest portion of the stratified epithelial cells was attached to the BM.

Scanning electron microscopy (SEM) provided detailed visualization of the surface ultrastructure of the skin in juvenile *A frenatus*. The skin surface was characterized by overlapping scales bearing posteriorly oriented ctenii, partially covered by the epithelial layer (EPL). In some regions, the EPL was removed during sample preparation, revealing underlying annuli (ans) (Figure 3A). Damaged areas of the epidermis (EPD) exposed epithelial cells (EPCs) and dense arrays of microridges (MRs), with annular structures clearly visible (Figure 3B). A lateral view of the trunk skin showed the multi-layered arrangement of the integument, with EPD and EPL overlaying the basement membrane (BM) and underlying dermis (DM), which contained fibroblasts (fb) (Figure 3C). The apical surface of intact EPD exhibited pronounced MRs, and the putative apical openings of goblet cells (ogbc) were discernible between the ridges (Figure 3D). Stratified EPC and capillaries (cp) derived from dermal vasculature were also observed in association with MRs in the upper EPD (Figure 3E). At the basal end, epithelial cells were closely adhered to the BM, indicating structural cohesion within the epidermal layers (Figure 3F). These morphological features reflect the specialized skin organization in *A. frenatus*.

### 2.4. Sequence Quality Control and Transcriptome Assembly

RNA-Seq analysis was employed as a high-resolution approach to identify and predict the gene regulatory networks driving the developmental metamorphosis of *A*. *frenatus*. To capture the full transcriptomic landscape, we sequenced six cDNA libraries, representing four distinct developmental stages: hatching (0 dph), 14 dph, 23 dph, and 45 dph (Figure S1). As detailed in Table S1, the experimental design incorporated stage-specific sample pooling, including decapitated trunks from 48 larvae at 0 dph, skin tissue from two biological pools of 48 larvae at 14 dph, and equimolar skin RNA pools from eight individual fish each at 23 and 45 dph. Sequencing on the Illumina HiSeq platform yields a robust dataset with high base-calling accuracy, with over 95% of bases meeting the Q30 quality standard (Phred score > 30) (Figure S2). The Sequence Quality Histograms demonstrated that mean quality scores consistently remained within the high-confidence green zone across all base positions, indicating minimal base-calling errors toward the ends of the reads (Figure S3). The total sequence yield was robust, ranging from approximately 36.7 million reads for hatching samples to 46.8 million reads for 14 dph samples. Read lengths averaged 100 bp paired-end for hatching (AF-Hat-1, AF-Hat-2) and 14 dph (AF-14d-2-1, AF-14d-2-2) samples, whereas the 23 dph (AF-23d) and 45 dph (AF-45d) samples yielded single-end read lengths of 52–53 bp, consistent with the specialized library preparation protocols used at these later stages (Table S1 and Figure S4). Unique mapping rates to the reference assembly remained exceptionally high across all libraries, ranging from 78.4% at hatching to 88.6% at 23 dph (Table S1 and Figure S5B). Analysis of Sequence Counts revealed that while unique read proportions were stable, the 23- and 45-dph libraries exhibited higher sequence duplication levels than the hatching and 14-dph libraries (Figure S5). Because PCR duplication and library complexity were not independently evaluated, these differences were treated as descriptive QC observations without biological interpretation. These metrics confirmed that the libraries possessed sufficient complexity for robust de novo assembly and quantification.

As *A*. *frenatus* is a non-model species, a de novo assembly was generated using the Trinity assembler to create a high-quality reference. The Non-redundant Unigenes Assembly Statistics indicated successful reconstruction, yielding a total of 31,054 non-redundant unigenes with a GC content of 49% (Figure S6). The assembly demonstrated high contiguity, as evidenced by an N50 of 4123 bp, ensuring that most of the transcriptome is represented by large contigs suitable for full-length CDS prediction. The Non-redundant Unigenes Length Distribution further validated the assembly quality, showing a significant concentration of transcripts exceeding 3000 bp (Figure S7). The average contig length was 2683 bp, with a median length of 1939 bp. This robust transcriptomic backbone provided the necessary resolution to map reads from each developmental stage and accurately quantify differentially expressed genes (DEUs) involved in the metabolic and immunological shifts identified in this study.

### 2.5. Transcriptomic and Functional Pathway Dynamics Across Developmental Stages

The global transcriptomic integrity and relationships among the six RNA-seq samples were evaluated by assessing dataset consistency and developmental stage-specific clustering. Principal component analysis (PCA), Pearson correlation matrices, and hierarchical clustering were applied to the global transcriptomic profiles, and the results are presented in Figure S8. To investigate the molecular shifts occurring during the metamorphosis of *A*. *frenatus*, differential expression analysis was performed using edgeR v3.22.5 with FDR correction to ensure statistical stringency. Using TransDecoder, the most likely ORFs were identified and translated into predicted protein sequences. Subsequent GO and KEGG pathway annotations facilitated systematic functional and metabolic network analyses, accompanying skin color development from hatching to the juvenile stage in *A*. *frenatus*.

**Figure 4:**
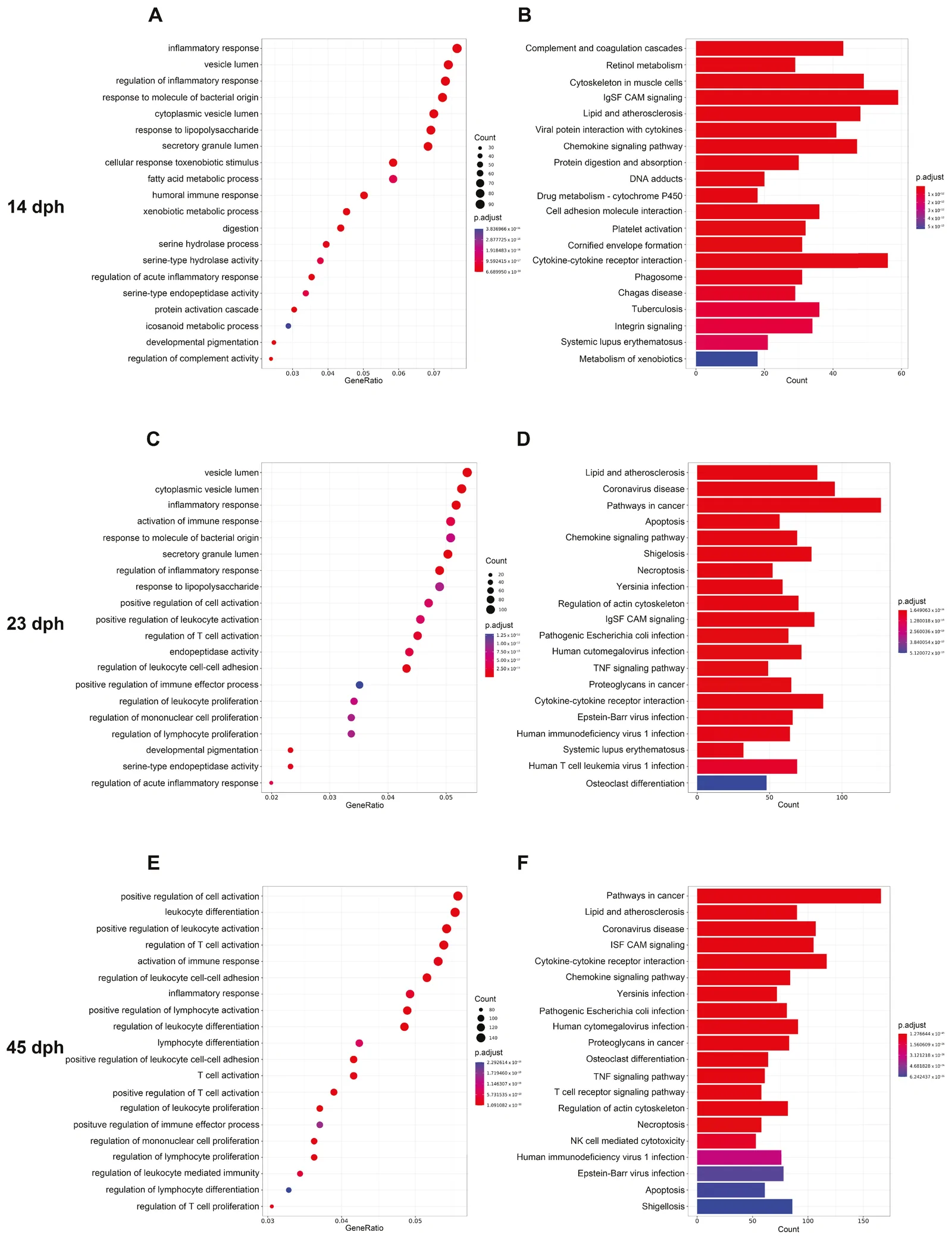
Functional Enrichment Analysis of Upregulated Unigenes Across Developmental Stages. This figure illustrates the functional profiles and metabolic pathways driven by significantly upregulated unigenes at 14 dph, 23 dph, and 45 dph relative to the hatching stage. (**A**,**C**,**E**) GO Enrichment Analysis: Dotplots representing the top biological process Gene Ontology (GO) terms for upregulated unigenes at 14 dph (**A**), 23 dph (**C**), and 45 dph (**E**). The *x*-axis indicates the GeneRatio, dot size reflects the unigene count, and the color scale corresponds to the statistical significance (FDR-adjusted *p*-value). (**B**,**D**,**F**) KEGG Enrichment Analysis: Barplots displaying significantly enriched KEGG pathways at 14 dph (**B**), 23 dph (**D**), and 45 dph (**F**). The *x*-axis represents the absolute number of unigenes (Count) mapped to each biological pathway. Early development (14 dph) is marked by prominent structural, digestive, and initial defense annotations, transitioning dynamically toward targeted cellular differentiation and tissue specialization by 45 dph.

The number of DEUs progressively increased as the larvae transitioned from hatching toward the juvenile stage. In the early phase at 14 dph, a marked transcriptomic change in the transcriptome was observed, with 2063 of 2667 unigenes upregulated relative to the hatching stage. These genes were predominantly annotated to GO categories related to innate defense, digestion, and developmental pigmentation. (Figure 4A). This was further supported by KEGG enrichment in pathways such as Complement and coagulation cascades, Retinol metabolism, and Protein digestion and absorption (Figure 4B). As the transition phase at 23 dph was reached, the number of upregulated genes increased to 3519, signaling a distinct shift toward the upregulation of transcriptomic profiles associated with leukocyte signaling. During this stage, immune-associated categories were enriched, with GO analysis identifying enrichment of immune-associated categories, including T-cell activation, leukocyte proliferation, and pigmentation (Figure 4C). KEGG enrichment included chemokine signaling and cytokine–cytokine receptor interaction pathways, suggesting developmental changes in the representation of immune-associated genes. However, these enrichment results do not independently demonstrate immune-cell recruitment or functional activation (Figure 4D). At 45 dph, annotated genes were predominantly associated with GO categories related to leukocyte differentiation and positive regulation of cell activation (Figure 4E). These enrichment patterns suggest developmental changes in immune-associated gene representation, although functional activation cannot be inferred from transcriptomic enrichment alone (Figure 4F).

**Figure 5:**
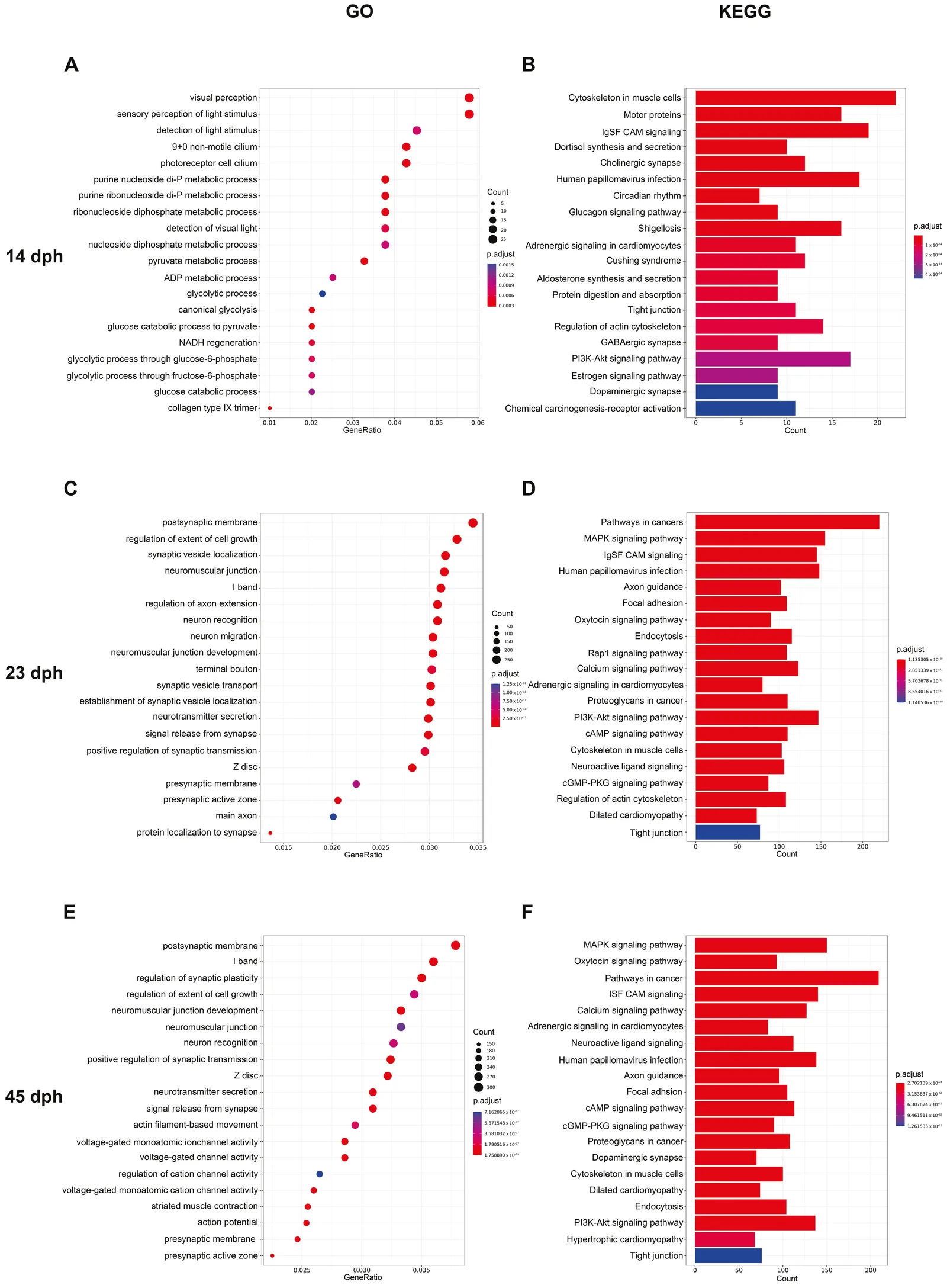
Functional Enrichment Analysis of Downregulated DEUs Across Developmental Stages. This figure illustrates the functional enrichment patterns among downregulated DEUs during post-hatch development at 14, 23, and 45 dph relative to the hatching stage. (**A**,**C**,**E**) GO Enrichment Analysis: Dotplots showing enriched GO biological processes for downregulated DEUs at 14 dph (**A**), 23 dph (**C**), and 45 dph (**E**). Notable terms include visual perception pathways during the early larval phase, progressing to a strong enrichment of structural and neuromuscular junction development components at later stages. The *x*-axis represents the GeneRatio, dot size reflects the unigene count, and the color scale corresponds to the adjusted *p*-value (FDR). (**B**,**D**,**F**) KEGG Enrichment Analysis: Barplots indicating significantly enriched KEGG pathways for downregulated DEUs at 14 dph (**B**), 23 dph (**D**), and 45 dph (**F**). The *x*-axis tracks the unigene count per pathway, showing stage-associated enrichment of pathways, including MAPK signaling and Axon guidance, among the downregulated DEUs at later developmental stages. These enrichment patterns indicate stage-associated differences in gene representation and should not be interpreted as direct evidence of functional pathway inactivation.

In contrast to the targeted upregulation of immune and mature metabolic pathways, the developmental transition was equally defined by downregulated unigenes also exhibited distinct stage-associated enrichment patterns. This downregulation reached its maximum scale at 23 dph and 45 dph, involving 10,706 and 10,284 DEUs, respectively. Early in development at 14 dph, a subset of 604 downregulated DEUs was identified, marking the commencement of sensory and glycolytic categories, which were enriched among downregulated DEUs, specifically targeting visual perception and canonical glycolysis (Figure 5A). This shift was reflected in the KEGG downregulation of Estrogen signaling and Dopaminergic synapse pathways (Figure 5B). The final stabilization into the juvenile phenotype was marked by the sustained downregulation of pathways associated with rapid cellular migration and organogenesis. At the later stages of 23 dph and 45 dph, the downregulated transcriptome was found to be heavily enriched in terms such as neuromuscular junction development, striated muscle contraction, and Z-disc components (Figure 5C,E). Correspondingly, KEGG analysis revealed that MAPK signaling, Calcium signaling, Axon guidance, and Focal adhesion pathways were enriched among the downregulated DEUs (Figure 5D,F). This broad repression suggests that the bioelectric signaling and rapid structural patterning required during the embryonic phase were represented by a reduced number of genes associated with developmental processes.

### 2.6. Discordance Between Transcript Abundance and Ribosome Occupancy and qPCR Validation of Pigmentation

**Figure 6:**
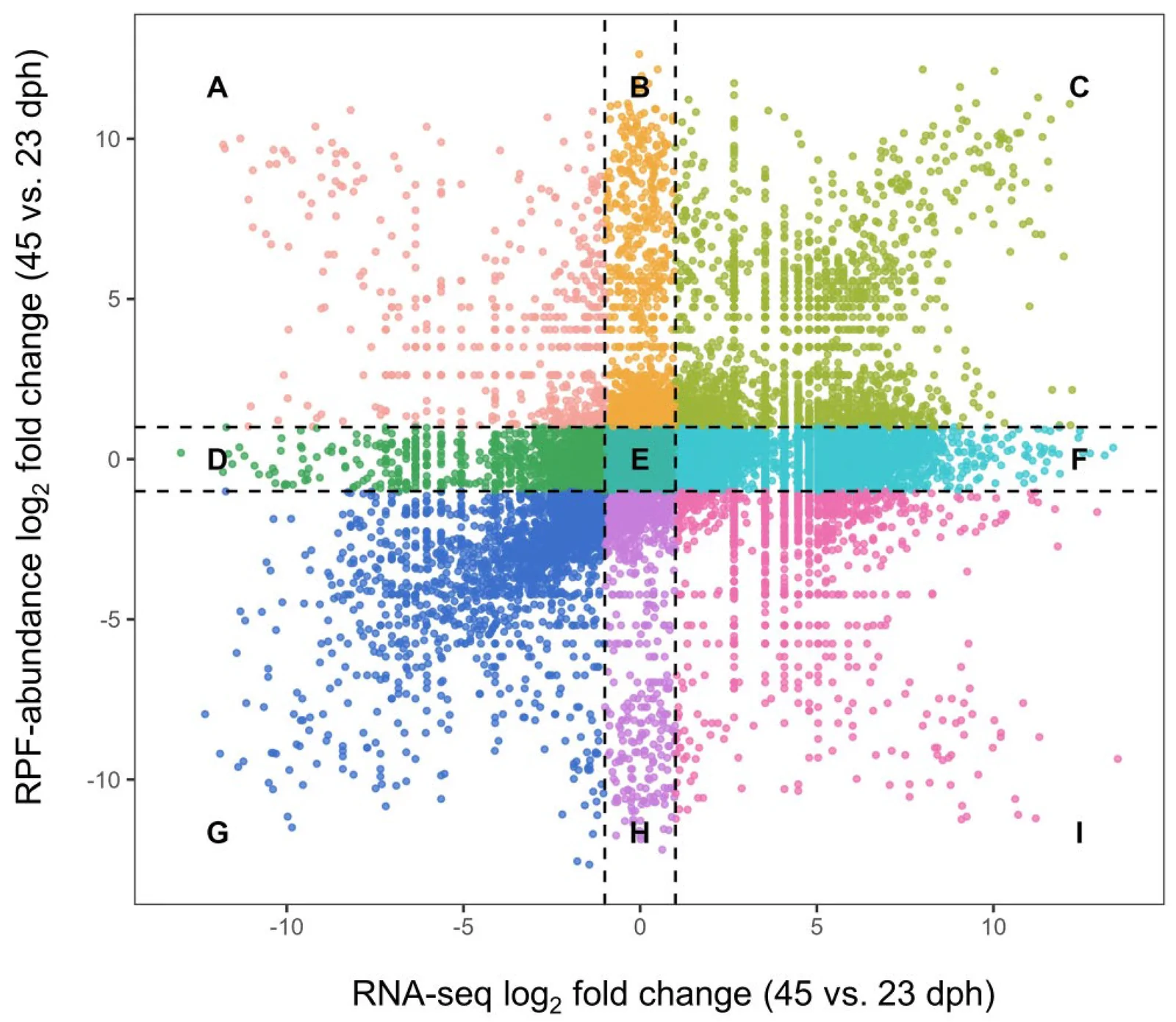
Integrated exploratory analysis of mRNAs and RPF abundance patterns during skin pigmentation (45 vs. 23 dph) in *A*. *frenatus*. Scatter plots show log_2_ fold changes in mRNA (*x*-axis) and RPF abundance (*y*-axis) levels. Genes are classified into nine exploratory regulatory categories (Groups A–I) based on expression trends, including concordant (Groups C,G) and discordant (e.g., Groups A,I) regulation. Dashed lines represent arbitrary fold-change thresholds (|log_2__FC| > 1) used for descriptive grouping rather than formal statistical significance testing. Group C highlights concordantly upregulated genes (*mchr1*, *rab38*, *myo5a*, *myo7a*, *pax6*, *pcna*, *p63*), which were further evaluated using independent biological replicate qPCR only for the verification of mRNA abundance levels.

To evaluate the coordination between mRNA and RPF abundance patterns during the pivotal stages of pigmentation in *A*. *frenatus*, we performed an integrated analysis of RNA-Seq and Ribo-Seq data comparing the 23 dph and 45 dph stages. The resulting nine-quadrant scatter plot illustrates the relationship between mRNA abundance (*x*-axis) and RPF levels (*y*-axis), providing a global view of the RNA and RPF-abundance landscape (Figure 6). Of the 31,054 non-redundant reference unigenes, 21,064 (67.83%) yielded numerical log_2_ fold-change values for both RNA and RPF abundance in the 45 vs. 23 dph comparison and were therefore retained for the exploratory integrated analysis (Figure 6). Based on the direction and magnitude of changes in RNA and RPF abundance, these unigenes were classified into nine groups (A–I) using |log_2_FC| > 1 as the categorical threshold. Group A contained 493 unigenes (2.34%) showing decreased RNA abundance but increased RPF abundance, whereas Group B comprised 1169 unigenes (5.55%) with relatively stable RNA abundance but increased RPF abundance. Group C included 2229 unigenes (10.58%) exhibiting concordant increases in both RNA and RPF abundance. Group D contained 1369 unigenes (6.50%) with decreased RNA abundance but relatively stable RPF abundance, while Group E, the largest category, comprised 7899 unigenes (37.50%) showing relatively limited changes in both RNA and RPF abundance. Group F included 3905 unigenes (18.54%) with increased RNA abundance but relatively stable RPF abundance. In contrast, Group G contained 1909 unigenes (9.06%) exhibiting concordant decreases in both RNA and RPF abundance, and Group H comprised 968 unigenes (4.60%) with relatively stable RNA abundance but decreased RPF abundance. Finally, Group I contained 1123 unigenes (5.33%) displaying increased RNA abundance accompanied by decreased RPF abundance. Together, these nine groups accounted for all 21,064 unigenes included in the integrated comparison and revealed a range of concordant and discordant RNA–RPF abundance patterns during the 23-to-45-dph developmental transition. Given the absence of biological replication in the Ribo-Seq dataset, these categories are interpreted as descriptive patterns rather than evidence of statistically significant translational regulation. Group C included several developmental and pigmentation-associated candidates, including *myc*, *pcna*, *p63*, *myo5a*, *myo7a*, *rab38*, and *mchr1*, which showed concordant increases in RNA and RPF abundance in the exploratory comparison.

**Figure 7:**
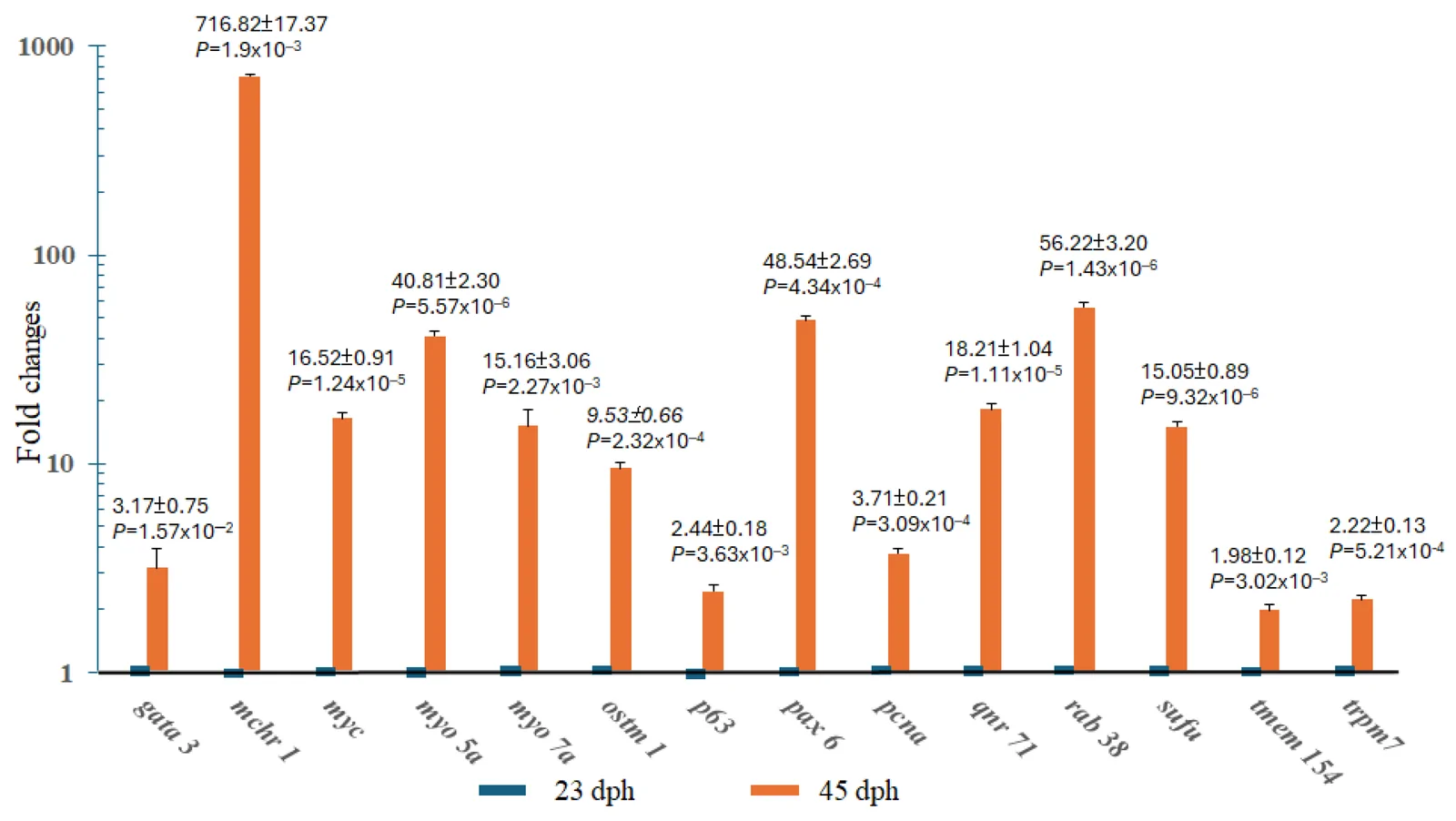
Quantitative real-time PCR validation of differentially expressed genes involved in skin maturation and pigmentation during the transition of juvenile stages in *A. frenatus.* Relative transcript levels of *gata3*, *mchr1*, *myc*, *myo5a*, *myo7a*, *ostm1*, *p63*, *pax6*, *pcna*, *qnr71*, *rab38*, *sufu*, *tmem154*, and *trpm7* were quantified by qPCR in skin tissues collected at 23 and 45 dph. Expression levels were normalized to *ef1a* and calculated using the 2^−ΔΔCt^ method, with expression at 23 dph set to 1. Bars represent the mean ± SEM (*N* = 8). The *y*-axis is presented on a log10 scale because gene expression spans nearly three orders of magnitude. Differences between developmental stages (23 vs. 45 dph) were analyzed using Welch’s two-sample *t*-test. *p*-values were adjusted for multiple testing using the Benjamini–Hochberg FDR procedure. An adjusted *p*-value < 0.05 was considered statistically significant.

To validate the transcriptomic datasets, the expression profiles of fourteen candidate genes associated with skin development, pigment cell differentiation, cell proliferation, and intracellular transport were quantified by qPCR at 23 and 45 dph (Figure 7). Overall, the genes examined exhibited significantly higher expression at 45 dph than at 23 dph, suggesting extensive molecular remodeling during juvenile skin development. Among all genes, *mchr1* exhibited the greatest expression, with an approximately 700-fold increase (*p* < 0.01), making it the most dramatically upregulated gene during this developmental transition. A second group of genes displayed markedly elevated expression, including *rab38* (~55-fold), *pax6* (~45-fold), and *myo5a* (~40-fold), all of which were highly significant (*p* < 0.001). Genes involved in cellular proliferation and developmental regulation, including *myc*, *myo7a*, *qnr71*, and *sufu*, exhibited moderate induction ranging from approximately 15- to 18-fold, whereas *ostm1* increased by approximately 10-fold. In contrast, genes associated with epithelial differentiation and tissue maintenance showed comparatively modest increases. *pcna* and *gata3* were elevated by approximately 3–4-fold, while *p63*, *tmem154*, and *trpm7* displayed only approximately two- to three-fold increases despite remaining statistically significant.

Notably, the magnitude of induction varied substantially among genes, spanning nearly three orders of magnitude on the logarithmic scale. This wide dynamic range suggests that the transition from 23 to 45 dph is driven by a subset of strongly activated regulatory genes rather than by uniform transcriptional activation across all components of the pigmentation and skin-development pathways. The exceptionally high expression of *mchr1*, together with the pronounced upregulation of *rab38*, *pax6*, and *myo5a*, highlights these genes as major molecular candidates associated with chromatophore maturation, pigment transport, and post-metamorphic skin differentiation.

## 3. Discussion

The developmental transition of *A*. *frenatus* from a translucent larva to a deep orange-red juvenile represents a complex physiological metamorphosis. By integrating a histological analysis with high-contiguity de novo transcriptomics and Ribo-Seq translatomics, we have characterized the coordinated, multi-tiered regulatory mechanisms governing this structural and phenotypic transformation.

### 3.1. Sequential Pigmentation During Clownfish Metamorphosis

The present observations demonstrate that pigmentation in *A*. *frenatus* is established through a stereotypic developmental sequence, with early melanophore differentiation followed by orange body pigmentation and the subsequent formation of the characteristic white cephalic bar. This progression agrees with recent studies showing that juvenile clownfish acquire their species-specific color pattern during metamorphosis rather than through continuous pigment accumulation [1,11]. White-bar formation is therefore considered a key developmental event accompanying the transition from the pelagic larval stage to the reef-associated juvenile stage [11].

The appearance of the white head bar at 45 dph in the present study is consistent with recent findings that clownfish white bars are formed by differentiated iridophores rather than leucophores [1]. Furthermore, thyroid hormone signaling has been identified as a central regulator of white-bar formation, coordinating pigment-cell differentiation with metamorphic development and ecological recruitment [11]. The developmental sequence observed in *A*. *frenatus* therefore supports the concept that pigment pattern formation is tightly integrated with endocrine-regulated metamorphosis in anemonefishes. Together, these observations establish a developmental framework for the subsequent histological and molecular analyses, allowing changes in skin architecture and gene expression to be interpreted in the context of clownfish-specific color pattern formation.

### 3.2. Histological Progression of Skin Development During Post-Hatch Growth

Histological examination revealed that the skin of *A*. *frenatus* undergoes progressive structural remodeling throughout post-hatch development (Figure 2). At 14 and 23 dph, the integument consisted of a thin epidermis directly overlying the musculature, while chromatophores were already present beneath the epidermal layer. These observations suggest that pigment cell differentiation is initiated before complete maturation of the dermis and scale structures [1,29,30]. The emergence of a distinct dermis, together with the first scale primordia at 45 dph, represents an important developmental transition in *A*. *frenatus*. Scale formation is widely recognized as a hallmark of juvenile skin maturation in teleosts and requires coordinated interactions among dermal fibroblasts, extracellular matrix components, and epidermal cells [3,31]. Recent transcriptomic studies further demonstrated that dermal cell populations actively regulate epidermal morphogenesis and provide positional cues that guide chromatophore organization during post-embryonic skin development [32]. Therefore, the simultaneous appearance of chromatophores and scale primordia observed in the present study suggests that dermal morphogenesis and pigment cell differentiation are tightly coordinated developmental events rather than independent processes.

Following metamorphosis, substantial structural remodeling was observed between 90 and 240 dph. The epidermis gradually became multilayered, the dermis expanded markedly, and overlapping scales were fully established. Comparable developmental processes have been described in zebrafish, medaka, and other teleosts, where epidermal stratification enhances barrier integrity while dermal expansion provides mechanical support for scale formation and pigment cell maintenance [3,30,32]. In addition, recent studies demonstrated that temporal regulation of ERK signaling controls epidermal proliferation and cellular hypertrophy during teleost skin development, thereby ensuring robust integumentary growth during juvenile stages [33]. These mechanisms may also contribute to the progressive epidermal thickening observed in *A*. *frenatus*.

The histological observations were accompanied by stage-associated transcriptomic changes in unigenes related to epidermal differentiation, extracellular matrix organization, cell proliferation, and pigmentation. The exploratory RNA–RPF comparison further identified candidate concordant and discordant abundance patterns during the transition from 23 to 45 dph. Although these molecular patterns cannot establish causal mechanisms underlying chromatophore differentiation or skin maturation, their temporal correspondence with the observed histological changes provides candidate molecular processes for further investigation [13,32,33]. Collectively, our histological analyses demonstrate that skin color formation in *A*. *frenatus* is a continuous developmental process involving epidermal stratification, dermal expansion, scale morphogenesis, and progressive accumulation of chromatophores. Scanning electron microscopy further revealed the mature organization of the juvenile skin, including overlapping ctenoid scales, stratified epithelial cells, abundant microridges, goblet-cell openings, and a distinct basement membrane separating the epidermis from the dermis. These structures characterize a functional teleost epidermis and contribute to epithelial integrity, mucus retention, mechanical protection, and host–environment interactions. Recent studies have further demonstrated that epidermal and dermal tissues actively coordinate skin morphogenesis by providing signaling environments that regulate epithelial differentiation, extracellular matrix remodeling, and pigment-cell organization during post-embryonic development [3,32]. Likewise, the establishment of microridges and epithelial stratification is an essential adaptation for maintaining the mucosal barrier and skin homeostasis in teleost fishes [3,30]. Together, these findings indicate that the acquisition of the characteristic skin architecture of *A*. *frenatus* is tightly integrated with pigment-cell development. This morphological framework provides an important basis for interpreting the stage-associated transcriptomic changes and exploratory RNA–RPF abundance patterns identified in the subsequent analyses.

### 3.3. Structural Maturation and Cutaneous Barrier Development

A notable finding of this study is the temporal alignment between the physical organization of dermal chromatophores and the structural maturation of cutaneous mucosal tissue. The enrichment of Chemokine signaling and Cytokine–cytokine receptor interaction pathways among upregulated DEUs at 23 and 45 dph coincided temporally with the histological organization of chromatophore-containing skin layers [3,9]. These signaling modules may also contribute to tissue remodeling and the spatial organization of chromatophores during dermal maturation, as cytokine- and chemokine-mediated signaling regulates cell migration, differentiation, and intercellular communication during vertebrate tissue development [1,3].

This developmental synchronization is further consistent with this pattern; functional categories associated with Natural killer cell-mediated cytotoxicity, T-cell activation, and leukocyte differentiation were represented among the upregulated DEU sets. These enrichment patterns suggest developmental changes in the representation of immune-associated transcripts but do not independently demonstrate maturation or functional activation of the corresponding immune pathways [34,35]. Members of the IgSF, including the polymeric immunoglobulin receptor (pIgR) and multiple antigen-recognition molecules, are essential mediators of cell adhesion, immune recognition, and mucosal immune communication [35,36]. The concurrent representation of immune-associated and pigmentation-related genes suggests that cutaneous maturation and pigment-pattern development occur over overlapping developmental periods [34,37].

qPCR further confirmed stage-specific expression differences in selected candidate genes associated with skin development and pigmentation. Rab38 is a key regulator of melanosome biogenesis and intracellular pigment vesicle trafficking, whereas MCHR1 participates in neuroendocrine regulation of pigmentation and physiological adaptation in vertebrates [38,39]. The stage-specific expression changes of selected pigmentation-related genes, together with enrichment of immune-associated functional categories, suggest that pigmentation and cutaneous maturation occur concurrently during juvenile development [3,38].

From this perspective, we hypothesize that the synchronization of chromatophore patterning and mucosal barrier development represents an integrated developmental program that simultaneously optimizes adaptive coloration and environmental protection during the critical transition from pelagic larvae to reef-associated juveniles.

### 3.4. Multi-Tiered Regulatory Control of the Juvenile Phenotype

The exploratory integration of RNA-Seq and Ribo-Seq data identified both concordant and discordant patterns between transcript and RPF abundance during skin color development in *A*. *frenatus* [26,40]. The predominance of Group C genes, which showed concordant increases in transcript and RPF abundance, indicates that increased transcript abundance was accompanied by increased RPF abundance for this group [41,42]. Specifically, the concordant RNA–RPF abundance patterns observed for pigmentation-associated candidates such as *myo5a*, *myo7a*, *rab38,* and *mchr1* identify these genes as candidates for further investigation of their regulation during juvenile pigmentation [35,38].

However, the discordant RNA–RPF patterns observed in Groups A, B, G, and H identify candidate genes for which changes in RPF abundance did not parallel changes in transcript abundance. Such patterns may reflect post-transcriptional regulation; however, this possibility cannot be established from the present unreplicated Ribo-Seq dataset [26,42]. Such transcription–translation uncoupling has increasingly been recognized as an important mechanism for buffering gene expression, optimizing protein production, and enabling developmental plasticity in vertebrates [40,42]. Likewise, the significant increase in *tmem154* expression observed by qPCR indicates a stage-specific change in transcript abundance, suggesting that tmem154 may be associated with skin development during this developmental transition.

At 45 dph, Axon guidance, MAPK signaling, and Tight junction pathways were represented among the downregulated DEU sets. These enrichment patterns indicate reduced representation of genes associated with these pathways relative to the earlier stage but do not demonstrate functional pathway inactivation [3,4]. During embryonic and larval development, elevated MAPK signaling and cell-adhesion pathways support cellular proliferation, migration, and tissue remodeling, whereas their subsequent attenuation is consistent with progressive tissue stabilization and functional maturation [3,4]. Accordingly, the transition to mature cutaneous architecture observed at 45 dph indicates successful progression from a vulnerable larval phenotype toward a physiologically stable juvenile stage.

Comparative transcriptomic and translatomic studies in teleosts, including *Oncorhynchus mykiss*, further demonstrate that developmental transitions are accompanied by coordinated transcriptional and post-transcriptional remodeling, reinforcing the view that post-transcriptional regulation represents a critical determinant of developmental phenotype beyond changes in mRNA abundance alone [40]. Together, these patterns identify stage-associated changes in transcript abundance and candidate RNA–RPF discordance that may provide targets for future investigation of post-transcriptional regulation during juvenile skin development.

### 3.5. Validation of Candidate Gene Expression Patterns by qPCR

The transition from larval to juvenile pigmentation in *A*. *frenatus* marks a critical developmental window characterized by extensive chromatophore differentiation, epidermal remodeling, and establishment of species-specific pigment patterns [4,35]. Independent qPCR analysis identified stage-specific transcript-abundance differences among the selected candidate genes between 23 and 45 dph [4,43].

Specifically, the increased expression of *myc* and *pcna* is consistent with their established roles in cell proliferation and may be associated with epidermal expansion and tissue remodeling during skin maturation [4,44]. The concurrent upregulation of *p63*, a master regulator of epidermal stem-cell maintenance and epithelial stratification, is consistent with the progressive maturation of the epidermis observed histologically [45]. In contrast, the increased expression of *pax6* and *gata3* may reflect continued activation of developmental transcriptional networks during juvenile skin maturation, suggesting broader roles in tissue patterning and differentiation rather than direct regulation of pigment-cell specification [4,43].

Genes involved in intracellular pigment transport, including *myo5a*, *myo7a*, and *rab38*, were also markedly upregulated at 45 dph. These proteins have established roles in melanosome biogenesis and intracellular pigment-organelle trafficking [46,47], making their increased transcript abundance consistent with the development of pigmentation during this period. Recent studies in zebrafish further demonstrate that proper pigment pattern formation depends on coordinated regulation of pigment-cell differentiation, migration, and interactions with the surrounding skin microenvironment during post-embryonic development [4,43].

Among all candidate genes, *mchr1* exhibited the greatest induction during the developmental transition. Melanin-concentrating hormone (MCH) signaling is a major neuroendocrine pathway that regulates physiological color change in teleosts, and emerging evidence suggests that it also contributes to long-term chromatophore physiology and pigmentation plasticity [32,48]. Therefore, the remarkable induction of *mchr1* observed in the present study may reflect increased neuroendocrine regulation accompanying chromatophore maturation and juvenile color stabilization.

Collectively, these qPCR results provide independent statistical support for stage-specific transcript-abundance differences in the selected candidate genes between 23 and 45 dph. The markedly different magnitudes of expression change among these candidates highlight *mchr1*, *rab38*, *pax6*, *myo5a*, and other responsive genes as candidates associated with juvenile pigmentation and skin development. Importantly, these qPCR results validate transcript abundance only and do not provide biological replication for the genome-wide RNA-Seq analysis or validation of RPF abundance and translational regulation.

### 3.6. Integrated RNA-Seq/Ribo-Seq Analysis During Skin Pigmentation

The exploratory integration of RNA-Seq and Ribo-Seq data identified both concordant and discordant relationships between transcript abundance and RPF abundance during the transition from 23 to 45 dph. These patterns provide candidate evidence for potential post-transcriptional regulation but, because only one pooled Ribo-Seq library was available per stage, they cannot establish statistically significant translational regulation. Several genes displayed discordant RNA–RPF abundance patterns, identifying candidates for potential post-transcriptional regulation. Such transcriptional–translational uncoupling has been demonstrated in biologically replicated studies of other developmental systems [25,41,49]; however, the present dataset does not permit formal testing of translational efficiency. Recent advances in ribosome profiling have further demonstrated that translational regulation is a fundamental mechanism that controls developmental progression, cellular differentiation, and tissue remodeling across diverse vertebrate systems.

The exploratory transcriptomic and translatomic dynamics observed in the present study likely reflect the rapid morphological and physiological remodeling associated with juvenile skin maturation. During this developmental period, epidermal stratification, chromatophore differentiation, extracellular matrix remodeling, and scale formation occur concurrently, requiring precise temporal coordination of protein synthesis. The temporal coincidence between these exploratory RNA–RPF patterns and morphological skin development provide a basis for future studies examining whether post-transcriptional mechanisms contribute to epidermal stratification, chromatophore differentiation, extracellular matrix remodeling, and scale formation. Similar transcriptional–translational discordance has recently been reported in rainbow trout, where integrated RNA-seq and ribosome profiling demonstrated that translational regulation substantially contributes to physiological adaptation independently of transcriptional responses [40]. Likewise, recent studies in zebrafish have shown that selective translational control governs developmental transitions by modulating protein production through differential ribosome occupancy and 5′-untranslated region (UTR)-mediated regulation, rather than by altering transcript abundance alone [46,50]. A methodological limitation of the present high-throughput sequencing screening is that, to overcome constraints in tissue yield during late metamorphosis, skin samples from eight individual fish per stage were pooled into single representative libraries for 23 and 45 dph (Tables S1 and S2). While the single-library Ribo-Seq comparisons provide an exploratory view of stage-associated RPF abundance patterns, qPCR analysis using eight individual biological replicates per stage (*n* = 8) provided independent statistical support for the expression patterns of selected candidate genes. These qPCR results validate transcript abundance only and do not address biological variability in the Ribo-Seq dataset.

Together, the integrated analysis identified concordant and discordant RNA–RPF abundance patterns during juvenile skin development in A. frenatus. These patterns provide a hypothesis-generating framework for identifying candidate genes that may be subject to post-transcriptional regulation, but they should not be interpreted as evidence of statistically validated translational regulation. Future studies incorporating independent biological Ribo-Seq replicates, formal translational-efficiency models, and protein-level validation will be required to determine the contribution of translational regulation to skin color formation in *A*. *frenatus*.

### Study Limitations

This study has several limitations that should be considered when interpreting the transcriptomic analyses. First, RNA-Seq at 23 and 45 dph was performed using one representative pooled RNA library per developmental stage, precluding estimation of stage-specific biological variation. Consequently, differential expression and GO/KEGG enrichment analyses involving these stages should be regarded as exploratory and hypothesis-generating rather than definitive statistical evidence of pathway activation. Second, comparisons between hatching larvae and later developmental stages may also reflect differences in sampled tissues, because decapitated trunks were necessarily used at hatching whereas skin tissue was analyzed after skin differentiation. Third, sequencing layouts differed between early and later developmental stages owing to differences in library preparation strategies. The present Ribo-Seq dataset was intended primarily as an exploratory complement to the transcriptomic analysis. Because only one representative pooled Ribo-Seq library was available for each developmental stage, formal statistical analyses of translational efficiency were not performed. In addition, several quality-control metrics commonly reported for Ribo-Seq datasets, including three-nucleotide periodicity, P-site offsets, metagene profiles, coding-sequence enrichment, and rRNA/tRNA contamination analyses, were not evaluated. Therefore, the observed discordance between transcript abundance and ribosome-protected fragment abundance should be interpreted as identifying candidate post-transcriptional regulatory events rather than definitive evidence of translational regulation. Future studies incorporating biological replication and comprehensive Ribo-Seq quality assessment will further clarify the contribution of translational regulation to skin pigmentation development. Although these factors may influence direct quantitative comparisons across stages, the integrated RNA-Seq, Ribo-Seq, histological, and qPCR analyses consistently identified candidate genes and biological processes associated with skin pigmentation development in *A. frenatus*. Future studies using independent biological RNA-Seq replicates across all developmental stages will further strengthen these observations.

## 4. Materials and Methods

### 4.1. Experimental Fish and Husbandry

Adult tomato clownfish (*A*. *frenatus*) were maintained in 50-L glass aquaria containing the host sea anemone *Heteractis magnifica*. A total of 10 independent breeding pairs were maintained under controlled laboratory conditions at 27 ± 1 °C with a salinity of 34 ± 1 psu and a 9 h light:15 h dark photoperiod. Adult fish were fed ad libitum twice daily with a mixed diet consisting of fresh shrimp, squid, and a commercial marine fish feed (Grobest Co., Ltd., Kaohsiung, Taiwan). Spawning activity of the 10 breeding pairs was monitored daily. Larvae produced by the 10 breeding pairs were combined to establish a common larval population. At both 0 and 14 dph, 96 larvae were sampled from this population and randomly allocated into two non-overlapping pools (48 larvae per pool), with no individual represented in both pools. RNA extraction and library preparation were performed separately for each pool, generating two separate pooled RNA-Seq libraries at each developmental stage. Thus, the two pools were independently processed from the pooling and RNA-extraction steps onward but originated from the same common larval population and were not considered independent biological replicates at the parental-pair or clutch level. Larvae were initially fed enriched rotifers (*Brachionus plicatilis*) and subsequently transitioned to copepods as development progressed. All experimental procedures involving animals were approved by the Institutional Animal Care and Use Committee (IACUC) of the Eastern Marine Biology Research Center, Fisheries Research Institute, Taiwan (Approval No. EMBRC-IACUC-108-005), and were conducted in accordance with institutional guidelines for the ethical use of experimental animals.

### 4.2. Skin Histology and Ultrastructural Analysis

For histological examination, five fish were randomly collected at 14, 23, 45, 90, 140, and 240 dph. Fish were euthanized using buffered tricaine methanesulfonate (MS-222; 500 mg/L), fixed in Bouin’s solution for 24 h, dehydrated through a graded ethanol series, embedded in paraffin, and sectioned at a thickness of 5 μm. Tissue sections were stained with hematoxylin and eosin (H&E) following standard histological procedures and examined using a light microscope (Olympus BX53, Olympus Corporation, Tokyo, Japan). For each developmental stage, skin sections from five individual fish were examined independently. Multiple microscopic fields were evaluated from each fish, and representative fields were selected based on section integrity, tissue orientation, staining quality, and the presence of histological features consistently observed among the examined individuals. For SEM, skin samples from five fish at 45 dph (approximately 10 mm total length) were fixed overnight in 2.5% glutaraldehyde prepared in 0.1 M phosphate buffer (pH 7.2) at 4 °C. Samples were post-fixed in 1% osmium tetroxide, dehydrated through a graded ethanol series, critical-point dried using liquid CO_2_, mounted on aluminum stubs, sputter-coated with gold, and observed using a scanning electron microscope (Hitachi S-3400N, Hitachi High-Technologies Corp., Tokyo, Japan). Putative structural identities in the SEM images were assigned based on characteristic morphology, anatomical location, and spatial relationships with adjacent tissues, together with comparison with the corresponding histological observations. The structure designated as the basement membrane (BM) appeared as a thin, continuous interface immediately beneath the epidermis at the epidermal–dermal boundary. Fibroblasts (Fb) were assigned based on elongated or spindle-shaped cellular profiles with processes located within the dermal connective-tissue matrix. Capillaries (Cap) were identified as small, thin-walled tubular structures with a discernible lumen within the dermal compartment. These assignments represent morphology-based identifications rather than ultrastructural or molecular confirmation.

### 4.3. RNA Extraction, Library Preparation, and Transcriptome Sequencing

To investigate gene expression during skin development, RNA samples were collected from larvae at 0, 14, 23, and 45 dph. At 0 dph, two independent pooled biological samples were prepared, with each pool consisting of decapitated 48 newly hatched larvae. At 14 dph, skin tissues were dissected under a stereomicroscope from two independent pools, each consisting of 48 larvae. At 23 and 45 dph, skin tissues were collected individually from eight fish at each developmental stage and pooled in equimolar ratios to generate a single representative RNA library for each stage. Because only one pooled RNA-Seq library was available for each of these developmental stages, subsequent transcriptomic analyses involving 23 and 45 dph should be regarded as exploratory and hypothesis-generating rather than formal statistical inference of stage-specific biological variation (Figure S1 and Table S1). Total RNA was extracted using TRIzol™ Reagent (Invitrogen, Carlsbad, CA, USA) following the method of Chomczynski and Sacchi [51]. RNA concentration and purity were determined using a NanoDrop spectrophotometer (Thermo Fisher Scientific, Wilmington, DE, USA), and RNA integrity was assessed using an Agilent 2100 Bioanalyzer (Agilent Technologies, Santa Clara, CA, USA). Only samples with A260/A280 > 2.0 and RNA integrity number (RIN) values between 6.8 and 10.0 were used for subsequent analyses. Poly(A)+ RNA was isolated using oligo(dT)-conjugated magnetic beads and fragmented prior to first-strand cDNA synthesis. Double-stranded cDNA libraries were prepared following the standard Illumina library construction protocol, including end repair, adapter ligation, PCR enrichment, and library quality assessment. Sequencing was performed on an Illumina HiSeq platform (Illumina Inc., San Diego, CA, USA). Details regarding sequencing layouts, read lengths, and mapping statistics across all stages are summarized in Table S1.

### 4.4. Ribosome Profiling (Ribo-Seq) Library Preparation

Ribosome profiling libraries were prepared according to the protocol described by Ingolia et al. [52] with minor modifications. At each developmental stage (23 and 45 dph), skin tissues collected from eight fish were combined to generate a single pooled biological sample. Following homogenization, the pooled tissue material was divided into two portions: one portion was used for total RNA extraction and subsequent RNA-Seq analysis, whereas the other was used for the isolation of ribosome-protected fragments (RPFs) and Ribo-Seq library preparation. Thus, the corresponding RNA-Seq and Ribo-Seq libraries at each developmental stage were derived from the same pooled biological source material. Fresh skin tissues collected at 23 and 45 dph were immediately lysed in ice-cold polysome lysis buffer containing 20 mM Tris-HCl (pH 7.4), 150 mM NaCl, 5 mM MgCl_2_, 1 mM dithiothreitol (DTT), 200 μg/mL cycloheximide, 1% Triton X-100, and 25 U/mL TURBO DNase I (Thermo Fisher Scientific, Waltham, MA, USA). Cycloheximide was included to arrest actively translating ribosomes and preserve ribosome–mRNA complexes. Cell lysates were digested with RNase I to remove unprotected RNA while preserving RPFs. RPFs were purified by polyacrylamide gel electrophoresis, size-selected for 27–40 nts, dephosphorylated using T4 polynucleotide kinase, and subjected to library preparation. For RNA-seq libraries, total RNA from the corresponding biological samples was fragmented and size-selected to obtain 25–65 nt RNA fragments. Following adapter ligation using T4 RNA Ligase 2 (truncated K227Q), first-strand cDNA synthesis was performed using SuperScript™ III Reverse Transcriptase (Invitrogen, Carlsbad, CA, USA), followed by single-stranded cDNA circularization using CircLigase™ (Lucigen, Middleton, WI, USA). Libraries were amplified with Phusion High-Fidelity DNA Polymerase (New England Biolabs, Ipswich, MA, USA), purified using AMPure XP beads (Beckman Coulter, Brea, CA, USA), and sequenced on an Illumina platform. Complete details regarding Ribo-Seq library yields, size selection parameters, Phred quality scores, and transcript mapping efficiencies across stages are summarized in Table S2 and Figure S9.

### 4.5. Bioinformatics Analysis and Integrated RNA-Seq/Ribo-Seq Analysis

All bioinformatic analyses were conducted under a CentOS 6.6 Linux operating system. Because *A. frenatus* lacks a complete reference genome, a de novo transcriptome was assembled using Trinity v2.3.2 [53]. Redundant transcript sequences were clustered at 95% sequence identity using CD-HIT v4.8.1 [54], resulting in a non-redundant reference transcriptome of 31,054 unigenes. Throughout this study, these non-redundant assembly units are referred to as “unigenes”. Unigenes were used as the reference units for expression quantification, differential-expression analysis, coding-sequence prediction, and functional annotation. Accordingly, differentially expressed unigenes are referred to as DEUs throughout the manuscript. The term “gene” is reserved for established or functionally annotated biological genes, whereas “transcript” is used when referring generally to RNA transcripts or transcript abundance. Protein-coding sequences were predicted using TransDecoder v3.0.1, and functional annotation was performed using the Trinotate pipeline v3.0.2 by integrating BLAST v2.2.31 homology searches, Pfam protein domain prediction, signal peptide prediction, and Gene Ontology annotation. Raw sequencing reads were processed using Cutadapt v5.2 [55] to remove adapter sequences and low-quality bases. After quality filtering and size selection (27–40 nts for Ribo-Seq and 25–65 nts for RNA-Seq; Table S2 and Figure S9), reads were mapped to the assembled transcriptome, and transcript abundance was quantified using RSEM v1.2.31 [45]. All libraries exhibited Q30 values greater than 95%, indicating high sequencing quality. Differential gene expression analysis was performed using the edgeR package v3.5 [47]. Raw read counts were normalized using the trimmed mean of M-values (TMM) method, and differential expression between developmental stages was evaluated using a false discovery rate (FDR)-adjusted *p*-value < 0.05 together with an absolute log_2_ fold-change (|log_2_FC|) > 1. For developmental stages represented by a single pooled RNA-Seq library (23 and 45 dph), differential expression and functional enrichment analyses were used primarily to identify candidate genes and biological processes. These analyses were interpreted descriptively because biological variability could not be estimated from singleton libraries. Functional enrichment analysis of DEUs was performed using Gene Ontology (GO, Version 10.3) and Kyoto Encyclopedia of Genes and Genomes (KEGG, release 111.0) annotations. The annotation background was defined as all successfully annotated unigenes in the non-redundant reference transcriptome with corresponding GO or KEGG functional annotations. Multiple testing was controlled using the Benjamini–Hochberg false discovery rate (FDR) procedure, and GO terms or KEGG pathways with an FDR-adjusted *p*-value < 0.05 were considered significantly enriched. To investigate exploratory transcriptional and post-transcriptional regulation during skin development, log_2_ FC values derived from RNA-seq and Ribo-seq datasets were integrated. Genes were classified into nine regulatory categories based on the direction and magnitude of changes in mRNA abundance and RPF levels using arbitrary fold-change thresholds ((|log_2_FC|) > 1), enabling the identification of concordant RNA and RPF regulation and candidate post-transcriptional regulatory events. Because one representative pooled Ribo-Seq library was available for each developmental stage, formal statistical analyses of translational efficiency were not performed. Instead, the integrated RNA-Seq/Ribo-Seq analysis was designed to descriptively identify concordant and discordant patterns between transcript abundance and ribosome-protected fragment (RPF) abundance, thereby generating candidate post-transcriptional regulatory events for future investigations.

### 4.6. Quantitative Real-Time PCR Validation

To independently assess selected transcript-abundance differences between 23 and 45 dph, quantitative real-time PCR (qPCR) was performed for candidate genes associated with skin development and pigmentation. Skin tissues were collected independently from eight fish at 23 dph and eight fish at 45 dph, providing eight biological replicates for each developmental stage. Total RNA was extracted individually from each fish and reverse-transcribed using SuperScript™ III First-Strand Synthesis SuperMix (Invitrogen, Carlsbad, CA, USA). Each qPCR reaction contained cDNA equivalent to 10 ng total RNA, 2× Power SYBR™ Green PCR Master Mix (Applied Biosystems, Foster City, CA, USA), and 200 nM each forward and reverse primer in a total reaction volume of 20 μL. Amplification was performed using an Applied Biosystems 7900HT Fast Real-Time PCR System under the following conditions: 95 °C for 10 min, followed by 40 cycles of 95 °C for 15 s and 60 °C for 1 min. Gene-specific primers were designed using Primer Express software v3.0 (Applied Biosystems) and synthesized by Mission Biotech Co., Ltd. (Taipei, Taiwan). Gene-specific primers used for qPCR are listed in Table 1. Each biological sample was analyzed in three technical replicates, and the average Ct value of the three technical replicates was used for subsequent analyses. Relative gene expression was calculated using the 2^−ΔΔCt^. method [56] with *translation elongation factor 1 alpha* (*ef1a*) as the internal reference gene. Expression at 23 dph served as the calibrator. qPCR experiments were performed in accordance with the MIQE guidelines [57]. Data are presented as the mean ± SEM of eight biological replicates (*N* = 8). The △Ct values from the 8 independent biological samples were used for statistical comparisons at each developmental stage. The 2^−ΔΔCt^ values are retained only for graphical presentation of relative expression. Differences between developmental stages (23 vs. 45 dph) were analyzed using Welch’s two-sample *t*-test. *p*-values were adjusted for multiple testing using the Benjamini–Hochberg False Discovery Rate (FDR) procedure. An adjusted *p*-value <0.05 was considered statistically significant.

## 5. Conclusions

This study describes the progressive development of skin architecture and pigmentation in *A*. *frenatus* from the larval to juvenile stages. Histological and SEM observations showed coordinated epidermal, dermal, scale, and pigment-cell maturation. Developmental RNA-Seq comparisons identified broad DEUs associated with pigmentation, tissue remodeling, metabolism, and immune-related annotations. The exploratory integration of RNA-Seq and Ribo-Seq data further identified genes displaying concordant or discordant RNA–RPF abundance patterns during the transition from 23 to 45 dph. Because the late-stage sequencing datasets were generated from one pooled library per stage, these patterns should be regarded as candidates for possible post-transcription rather than as statistically confirmed changes in translational efficiency. Independent qPCR analysis using eight fish per stage supported developmental changes in selected genes, including *mchr1*, *rab38*, *pax6*, and *myo5a*. Future studies incorporating biological Ribo-Seq replicates, formal translational-efficiency models, protein-level validation, transmission electron microscopy, and immunohistochemistry will be required to verify the proposed regulatory mechanisms and chromatophore identities.

## Figures and Tables

**Table 1:** Gene-specific primer sequences used in qPCR.

Gene	Forward Primer	Reverse Primer
*ef1a*	CCCTGCAGGACGTCTACAAAA	GGAGCAAAGGTGACGACCAT
*gata3*	TCAATCGGAGCAGGGTCATC	TCAGGCTACTTGGTGGGAAGA
*mchr1*	CGACATCACCACTTTCATGCA	CCAGCCCGTTGGCTATAACTC
*myc*	ACAGCGCCGCAACGAA	CTTCAGGATCACCACCTTGGA
*myo5a*	GCTACTGCGGGAGGCTAAGG	CTGCAGGTAGACGATGGAGTGA
*myo7a*	CAGCTGAGGTATTCTGGGATGAT	GGACTCTGTAGCGATCCACAAAC
*ostm1*	GGCCAGCGGTATGTGGACTAT	TGTCCACCAGTTTGCCATAAGT
*p63*	CGACTCACGGAAAAGTCAACAA	GTGAGGCCTCCAGACATGCT
*pax6*	GATGGCTGCCAACAACAAGAC	TCTTGAGCTGCAGTCGCATCT
*pcna*	AGCCCGTCCAGCTGATCTT	GGGATATCAGCCGACATGCT
*qnr71*	TCCTCCAGCATCACCATCAA	TCGCGCTTCCTGTAGACCAT
*rab38*	GCGGTTCGGCAACATGAC	TTTTGTGACGGCCTCAAAGG
*sufu*	ACCTAGTGGGTTTGGCTTTGAG	AGCCAGGCCTTGCATGAG
*tm* *em* *154*	AAGACCACAGCTCAGGACACAGT	GAGGTTCCGTCTGTGGCATT
*trpm7*	AAGTACGGCGCTGAGGTCAA	TGACCAATTCGCGCATGTAT

## Data Availability

The transcriptomic (RNA-Seq and Ribo-Seq) sequencing data generated in this study have been deposited in the NCBI Sequence Read Archive (SRA) under BioProject accession number PRJNA1284240. These data include samples from *A*. *frenatus* at different post-hatching stages and are publicly available. The dataset can be accessed via the following link: https://www.ncbi.nlm.nih.gov/bioproject/PRJNA1284240 (accessed on 8 June 2026).

## Supplementary Materials

The following supporting information can be downloaded at: https://www.mdpi.com/article/10.3390/ijms27188289/s1.
