# Supplementary material for: Integrated Transcriptomic Analysis of Skin Pigmentation Development in Amphiprion frenatus
Source: Int J Mol Sci. 2026 Sep 17;27(18):8289. doi: 10.3390/ijms27188289 (PMC13607702; doi:10.3390/ijms27188289)
Supplement: Supplementary file 1 [file ijms-27-08289-s001.zip › ijms-4395528-supplementary.pdf]

Supplementary Materials

Table S1. Summary of experimental design, sample pooling strategies, sequencing parameters, and alignment statistics for *A. frenatus* RNA-seq libraries across developmental stages.

Stage	Sample Name	Biological Unit / Tissue Collected	Number of Fish	Pooling Strategy	Biological Libraries (N)	Sequencing Layout	Read Length	Total Clean Reads	Unigene Assignment Rate (% of Mapped Reads)	Alignment Rate to Reference (% of Clean Reads)
**0 dph**	AF-Hat-1	Decapitated trunk tissue	48	48 trunks pooled	2	Paired-End (PE)	100 bp	24,785,102	78.4%	58.4%
**0 dph**	AF-Hat-2	Decapitated trunk tissue	48	48 trunks pooled		Paired-End (PE)	100 bp	21,502,314	78.4%	58.6%
**14 dph**	AF-14d-2-1	Dissected larval skin	48	48 skin samples pooled	2	Paired-End (PE)	100 bp	27,614,208	81.2%	59.6%
**14 dph**	AF-14d-2-2	Dissected larval skin	48	48 skin samples pooled		Paired-End (PE)	100 bp	27,908,116	81.2%	59.6%
**23 dph**	AF-23d	Dissected juvenile skin	8	8 skin samples pooled	1	Single-End (SE)	52–53 bp	14,482,910	88.6%	26.1%
**45 dph**	AF-45d	Dissected juvenile skin	8	8 skin samples pooled	1	Single-End (SE)	52–53 bp	21,120,404	86.8%	43.7%

Note: Clean read counts and mapping percentages directly reflect the RSEM and Bowtie 2 alignment statistics reported in Supplementary Figures S4, S5A–B, S6, S7A–B, and S8A–B.

Table S2. Summary of library metrics, size selection parameters, Phred quality scores, and unigene mapping statistics for *A. frenatus* ribosome profiling (Ribo-Seq) and parallel RNA-Seq across developmental stages.

Parameter / Metric	Ribo_AF23D	Ribo_AF45D	RNA_AF23D	RNA_AF45D
Input Tissue / Lysate Amount	48.3 µg	52.2 µg	17.5 µg (Cytosolic)	25.9 µg (Cytosolic)
RNA Integrity (RQN / RIN)	6.8	7.9	6.8	7.9
Raw Sequencing Reads	47,313,320	58,735,266	33,189,163	40,144,189
Reads After Adaptor Removal	46,250,536 (97.75%)	56,490,589 (96.18%)	14,834,768 (44.70%)	21,489,311 (53.53%)
Size-Selected Reads Sent to Aligner	44,885,679 (27–40 nts)	50,208,389 (27–40 nts)	14,540,460 (25–65 nts)	21,207,191 (25–65 nts)
Alignment to Unigene Reference (%)	43,469,241 (96.84%)	48,260,575 (96.12%)	11,549,973 (79.43%)	19,353,548 (91.26%)
Sequencing Platform	Illumina NextSeq 500	Illumina NextSeq 500	Illumina NextSeq 500	Illumina NextSeq 500

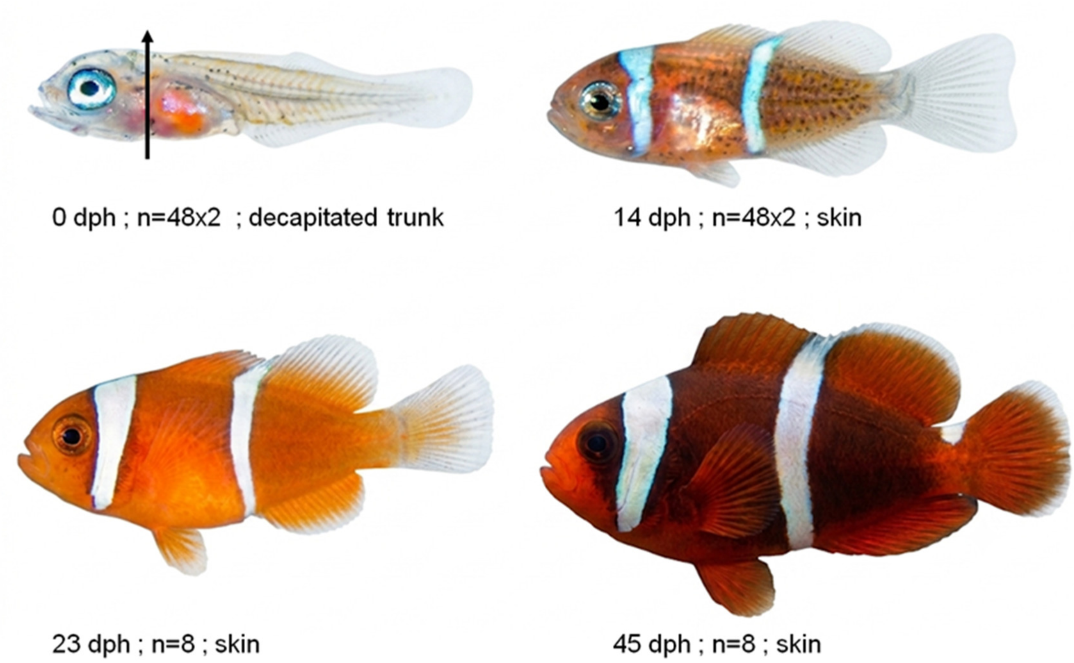

Figure S1. Experimental Sampling Strategy for RNA-seq and Ribo-seq Analyses during Skin Development of *A. frenatus*. Samples were collected at four representative developmental stages: newly hatched larvae (0 dph), pre-metamorphic larvae (14 dph), metamorphic juveniles (23 dph), and post-metamorphic juveniles (45 dph). At 0 dph, the skin could not be separated because of the small body size; therefore, decapitated trunk tissues (n = 48 larvae per biological replicate, two replicates) were collected for RNA sequencing (RNA-seq). At 14 dph, isolated skin tissues were collected from pooled larvae (n = 48 larvae per biological replicate, two replicates). At 23 and 45 dph, skin tissues were dissected individually from eight fish at each stage (n = 8). These samples were subsequently subjected to integratedRNA and RPF analyses to investigate molecular regulation during skin maturation and pigmentation.

Sample Summary

Sample Summary This table shows the summary information about samples.

Copy table | Configure Columns | Plot | Showing 6/6 rows and 7/7 columns.

Sample_Name	% GC	Average Read Length	% Bases Q ≥ 20	% Bases Q ≥ 30	Average Quality	# Reads	# Bases
Af-23d	44.80%	52.83	97.06%	95.47%	34.66	14,470,548	764,442,755
Af-45d	41.18%	51.57	97.16%	95.58%	34.68	21,139,023	1,090,090,055
Af-hat-2	47.57%	99.89	98.80%	95.28%	36.77	73,412,564	7,333,365,058
Af-hat-1	47.64%	99.88	98.86%	95.50%	36.84	84,954,938	8,485,647,129
Af-14d-2-2	47.45%	99.90	98.87%	95.56%	36.87	93,654,072	9,356,488,878
Af-14d-2-1	47.33%	99.91	98.87%	95.57%	36.88	92,441,026	9,235,505,578

Figure S2. Summary Statistics for RNA-Seq Libraries Across Developmental Stages of *A. frenatus*. The summary statistics for the six cDNA libraries represent the four developmental time points analyzed, specifically hatching (AF-hat-1, AF-hat-2), 14 dph (AF-14d-2-1, AF-14d-2-2), 23 dph (AF-23d), and 45 dph (AF-45d). All samples exhibited exceptional base-calling accuracy, with the percentage of bases meeting the Q30 quality standard consistently exceeding 95% and average quality scores ranging from 34.66 to 36.88. This high level of precision ensures the reliability of the reads utilized for downstream de novo transcriptome assembly and differential expression analysis. Data varied by stage, with the hatching and 14 dph samples producing deep coverage between 73.4 and 93.6 million reads, while the 23 dph and 45 dph libraries yielded approximately 14.5 million and 21.1 million reads, respectively. Furthermore, the average read lengths were approximately 100 bp for the hatching and 14 dph stages, compared to 52–53 bp for the later stages, while the GC content remained stable across all samples within a range of 41.18% to 47.64%.

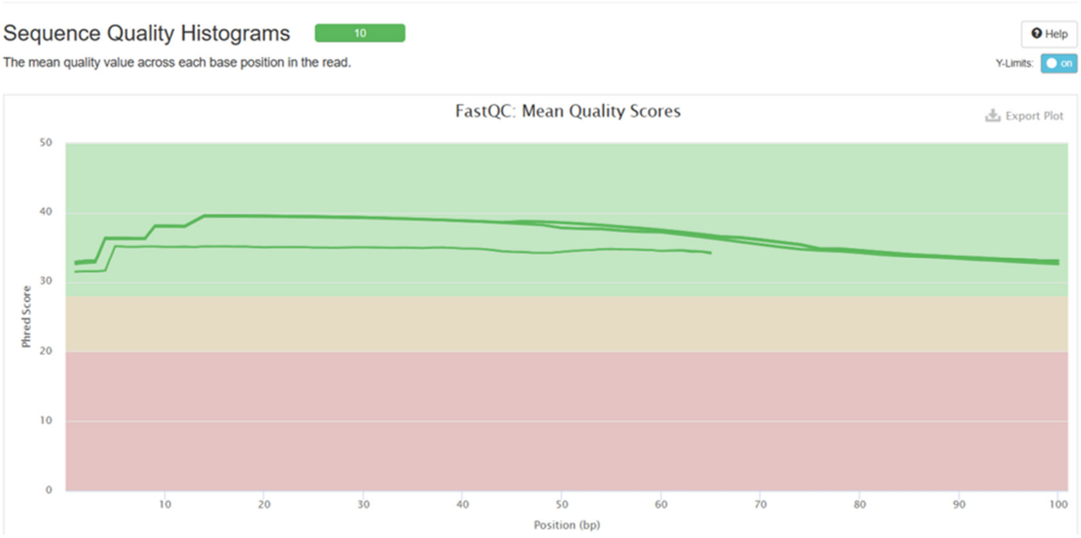

Figure S3. Sequence Quality Histograms across Base Positions. The mean quality scores (Phred scores) for the sequencing reads across each base position in the library are illustrated. The y-axis represents the Phred score, while the x-axis indicates the position along the read in base pairs (bp). Throughout the entire read length (up to 100 bp), the mean quality values remain consistently within the high-confidence green zone

(Phred score > 28), reflecting the exceptional reliability of the base calling and the high technical quality of the Illumina HiSeq sequencing libraries used for the *A. frenatus* transcriptome assembly.

General Statistics

Copy table | Configure Columns | Plot | Showing 10/10 rows and 3/5 columns.

Sample Name	% GC	Length	M Seqs
AF-14d-2-1.R1	46%	100 bp	46.2
AF-14d-2-1.R2	47%	100 bp	46.2
AF-14d-2-2.R1	47%	100 bp	46.8
AF-14d-2-2.R2	47%	100 bp	46.8
AF-23d.R1	44%	53 bp	14.5
AF-45d.R1	41%	52 bp	21.1
AF-Hat-1.R1	47%	100 bp	42.5
AF-Hat-1.R2	47%	100 bp	42.5
AF-Hat-2.R1	47%	100 bp	36.7
AF-Hat-2.R2	47%	100 bp	36.7

Figure S4. General Statistics of Raw Sequencing Reads for *A. frenatus* Libraries. The foundational metrics for the ten sequencing data files (replicates and read pairs) across the four developmental stages: 14 dph (AF-14d), 23 dph (AF-23d), 45 dph (AF-45d), and hatching (AF-Hat) were summarized. The average GC content across all samples remained stable, ranging from 41% to 47%, which is consistent with typical teleost genomic profiles. Sequence length was standardized at 100 bp for the hatching and 14 dph libraries, while the 23 dph and 45 dph libraries consisted of 53 bp and 52 bp reads, respectively. Total sequence yield per file was robust, ranging from 14.5 million reads (M Seqs) in the 23 dph stage to 46.8 million reads in the 14 dph stage, providing the high-depth coverage required for accurate *de novo* transcriptome reconstruction and quantification.

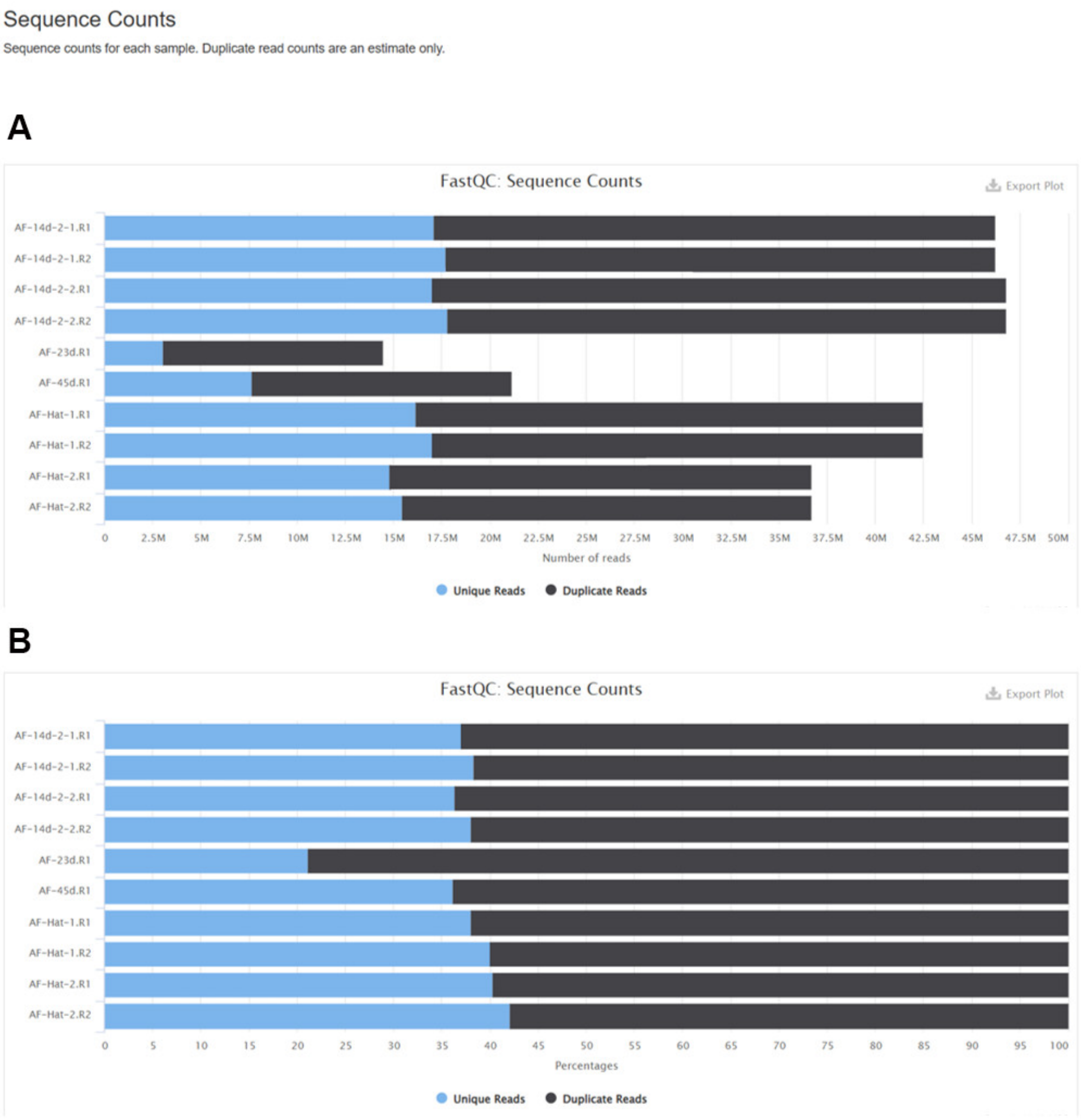

Figure S5. Distribution of Unique and Duplicate Sequence Counts across Libraries. FastQC analysis of sequence counts for each sample presented in absolute read numbers (A) and as percentages of total reads (B). Blue bars represent unique reads, while black bars indicate estimated duplicate read counts. Most libraries, particularly at the hatching and 14 dph stages, maintain a robust proportion of unique reads. The AF-23d and AF-45d libraries showed higher sequence-duplication levels than the 0- and 14-dph libraries. Because PCR duplication and library complexity were not independently evaluated, these differences are reported as descriptive sequencing-quality observations without biological interpretation.

Non-redundant Unigenes Assembly Statistics

Trinity.fasta : Raw non-redundant unigenes

Trinity.95.fasta : non-redundant unigenes by 95% similarity clustering <<< This is the final assembly (.fasta).

	Features	Values
1	Total trinity 'non-redundant unigenes':	31,054
2	Percent GC:	49
3	Contig N10:	9,246
4	Contig N20:	7,181
5	Contig N30:	5,891
6	Contig N40:	4,912
7	Contig N50:	4,123
8	Median contig length:	1,939
9	Average contig:	2,683

- N50 size: 50% of the genome is in contigs as large as the N50 value.

Figure S6. Non-redundant Unigenes Assembly Statistics for de *novo A. frenatus*. The foundational metrics for the final transcriptome assembly (Trinity.95.fasta), generated by clustering raw non-redundant unigenes at 95% similarity, are presented. The assembly successfully reconstructed a total of 31,054 non-redundant unigenes with an overall GC content of 49%. The quality and contiguity of the assembly are evidenced by an N50 size of 4,123 bp, meaning half of the total assembly length is contained in contigs of at least this size. Additionally, the assembly achieved a high average contig length of 2,683 bp and a median length of 1,939 bp, providing a robust reference for accurate CDS prediction and downstream functional annotation.

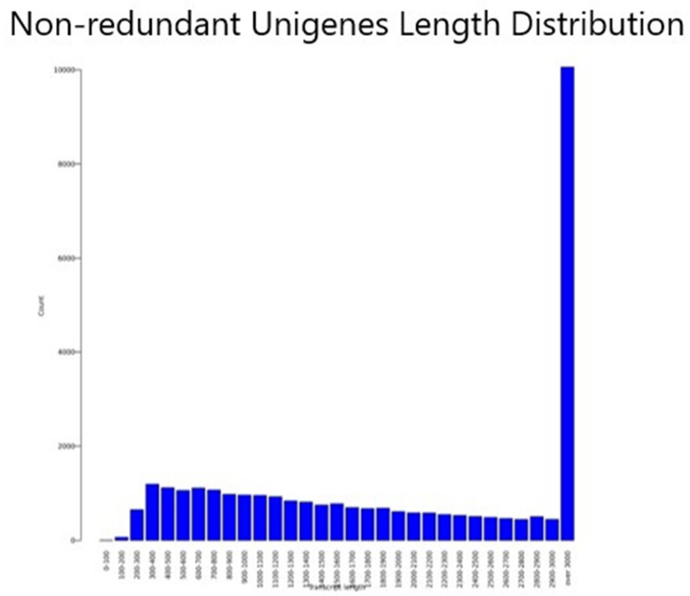

Figure S7. Non-redundant Unigenes Length Distribution of the *A. frenatus de novo* Assembly. The distribution of non-redundant unigenes lengths within the final assembly, which comprises a total of 31,054 non-redundant unigenes, was illustrated. The x-axis represents the non-redundant unigenes length categorized into 100 bp bins, and the y-axis indicates the total count of non-redundant unigenes within each size range. The distribution shows a broad representation of transcript sizes, with a substantial peak occurring in the "over 3000" bp category. This high concentration of long non-redundant unigenes correlates with the calculated N50 value of 4,123 bp, demonstrating the high contiguity of the assembly and its suitability for full-length protein prediction and detailed functional annotation.

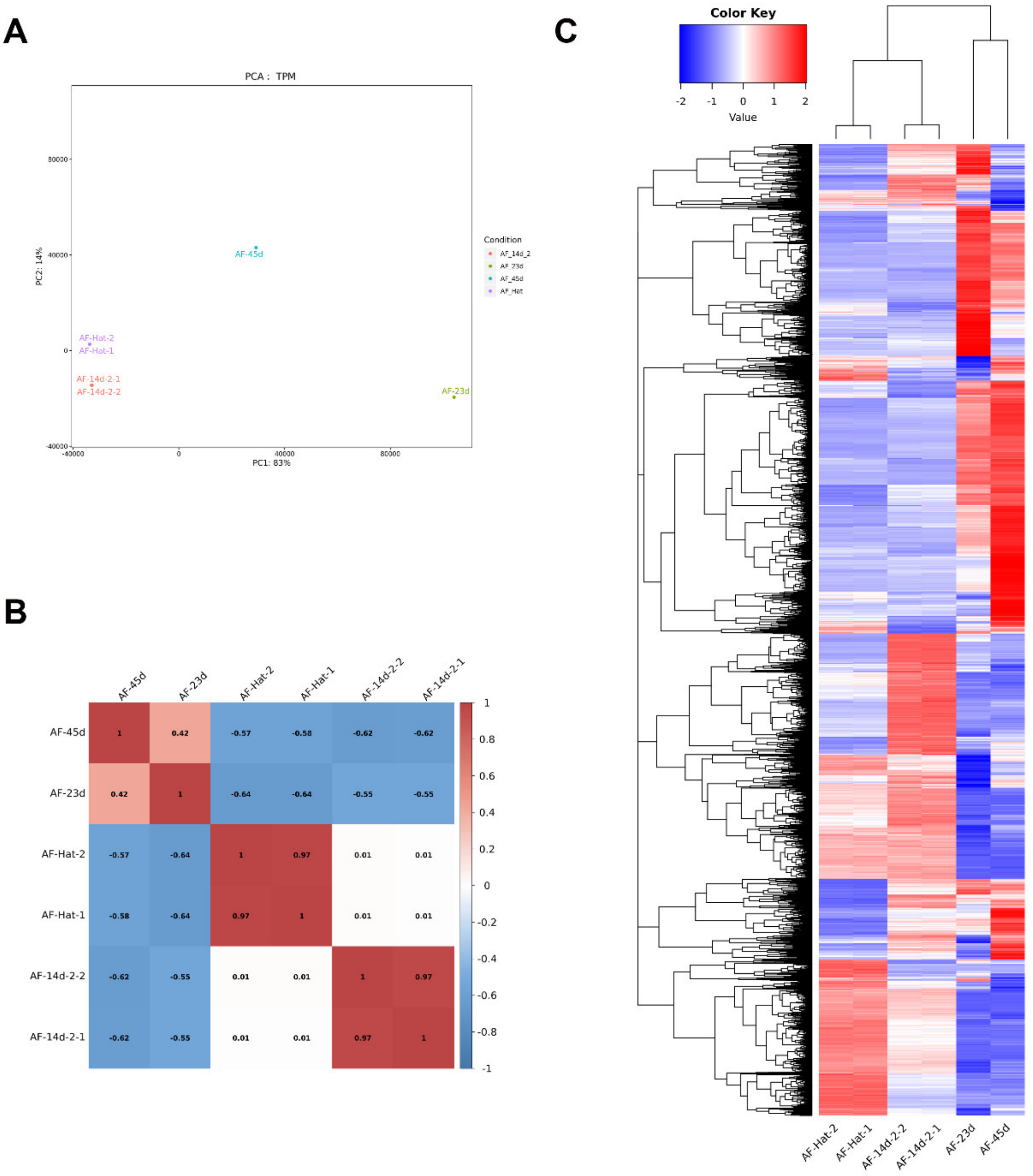

Figure S8. Global Transcriptomic Relationships and Sample Integrity. PCA based on Transcripts Per Million (TPM) values revealed distinct clustering of samples according to their developmental stages, with the first two principal components (PC1 and PC2) accounting for 97% of the total variance. PC1, which explained 83% of the variance, primarily separated the early stages of hatching and 14 dph from the later juvenile stages of 23 dph and 45 dph (Figure S8A). The reliability of the biological replicates was further confirmed through a Pearson correlation matrix, which demonstrated high intra-stage correlation coefficients of 0.97 for both hatching and 14 dph replicates (Figure S8B). In contrast, a negative correlation ranging from -0.55 to -0.64 was observed between early and late developmental stages, reinforcing the evidence of a distinct metabolic and physiological shift during metamorphosis. These relationships were further visualized through a global expression heatmap with hierarchical clustering, which grouped the samples into two primary clades: an early developmental

group comprising hatching and 14 dph samples, and a late developmental group containing 23 dph and 45 dph samples (Figure S8C). The heatmap effectively illustrated large blocks of stage-specific gene expression, where transcripts were highly active at the hatching and 14 dph stages and were largely repressed by 45 dph as a new set of juvenile-specific transcripts emerged. Collectively, these global patterns provide a robust foundation for the subsequent identification of stage-specific differentially expressed genes and their functional roles in the development of *A. frenatus*.

A.

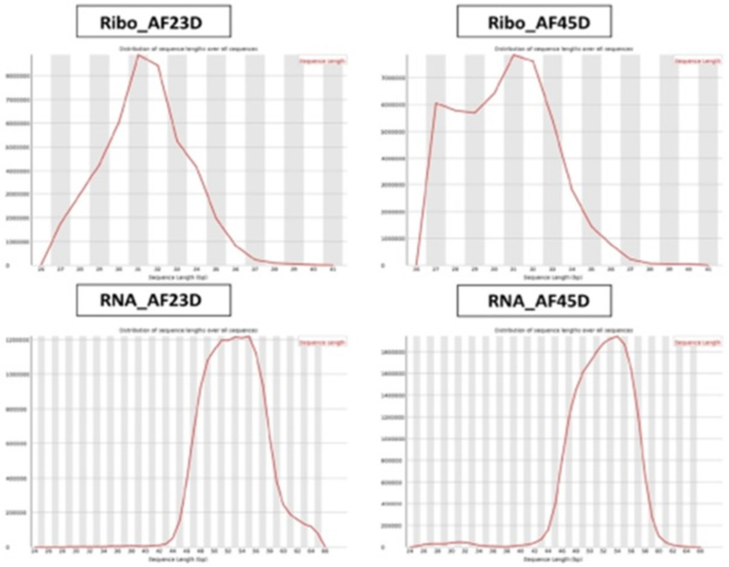
Ribo_AF23D
Ribo_AF45D
RNA_AF23D
RNA_AF45D

B.

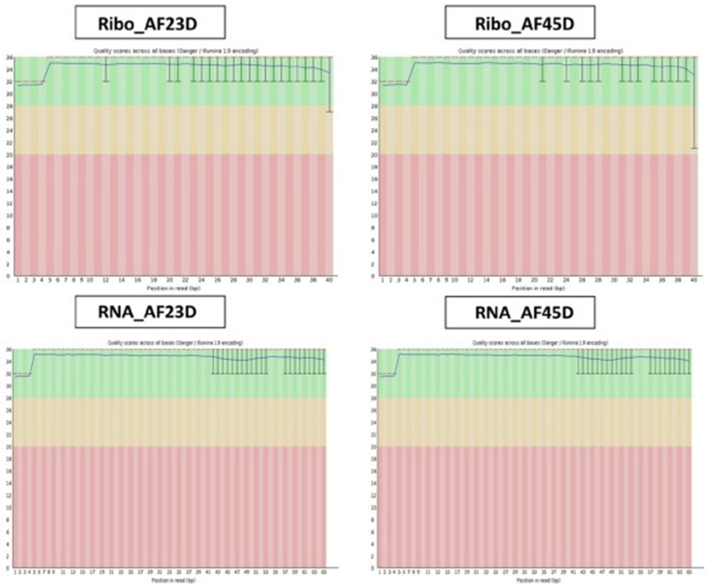
Ribo_AF23D
Ribo_AF45D
RNA_AF23D
RNA_AF45D

C.

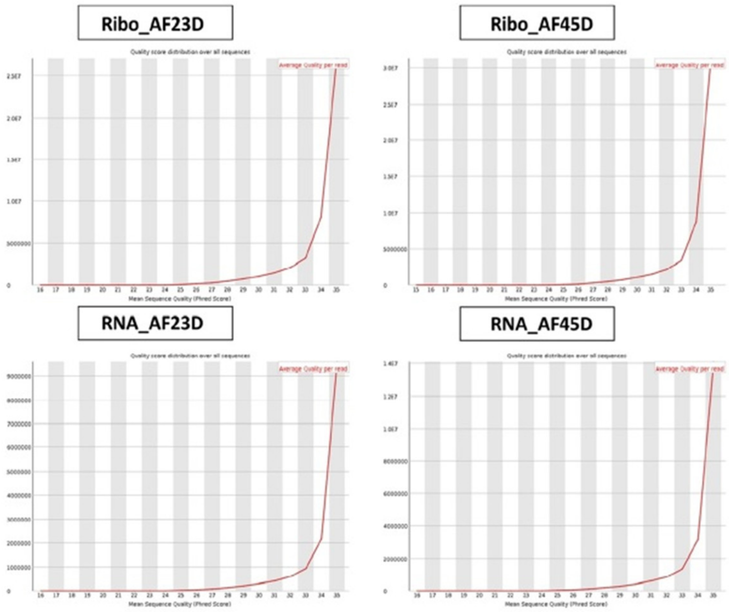
Ribo_AF23D
Ribo_AF45D
RNA_AF23D
RNA_AF45D

Figure S9. Quality control and read-length characteristics of Ribo-Seq and parallel RNA-Seq libraries from *A. frenatus*. (A) Fragment-length distributions showed enrichment of Ribo-Seq reads within 27–40 nucleotides, with a sharp peak at approximately 31–32 nucleotides consistent with monosome-protected ribosome footprints, whereas RNA-Seq reads retained within 25–65 nucleotides displayed a broader peak at approximately 52–54 nucleotides, reflecting the fragmented RNA population. (B) Per-base sequence quality profiles for Ribo_AF23D, Ribo_AF45D, RNA_AF23D, and RNA_AF45D remained predominantly within the high-quality range across the retained read positions, with most bases exhibiting Phred scores above 30. (C) Per-sequence quality distributions further showed that most reads in all four libraries had high mean Phred quality scores, with the distributions strongly concentrated near 34–35, supporting the overall reliability of the sequencing data for downstream transcriptomic and Ribo-seq analyses.
